# Mixed mechanism of conformational selection and induced fit as a molecular recognition process in the calreticulin family of proteins

**DOI:** 10.1371/journal.pcbi.1010661

**Published:** 2022-12-12

**Authors:** Ashalatha Sreshty Mamidi, Avadhesha Surolia

**Affiliations:** Molecular Biophysics Unit, Indian Institute of Science, Bangalore–India; University of Maryland School of Pharmacy, UNITED STATES

## Abstract

The fundamental question on the mechanism of molecular recognition during ligand binding has attracted a lot of scientific scrutiny. The two competing theories of ligand binding–“induced fit” and “conformational selection” have been proposed to explain biomolecular recognition. Since exploring a family of proteins with similar structural architectures and conserved functional roles can provide valuable insight into the significance of molecular structure and function, we performed molecular dynamics simulations on the calreticulin family of proteins, which specifically recognize monoglucosylated N-glycan during the protein folding process. Atomistic simulations of lectins in free and bound forms demonstrated that they exist in several conformations spanning from favorable to unfavorable for glycan binding. Our analysis was confined to the carbohydrate recognition domain (CRD) of these lectins to demonstrate the degree of conservation in protein sequence and structure and relate them with their function. Furthermore, we computed the lectin-glycan binding affinity using the mmPBSA approach to identify the most favorable lectin conformation for glycan binding and compared the molecular interaction fields in terms of noncovalent bond interactions. We also demonstrated the involvement of Tyr and Trp residues in the CRD with the non-reducing end glucose and central mannose residues, which contribute to some of the specific interactions. Furthermore, we analyzed the conformational changes in the CRD through SASA, RMSFs and protein surface topography mapping of electrostatic and hydrophobic potentials. Our findings demonstrate a hybrid mechanism of molecular recognition, initially driven by conformational selection followed by glycan-induced fluctuations in the key residues to strengthen the glycan binding interactions.

## Author summary

Conformational dynamics impact molecular recognition. Understanding the time dependent fluctuations in the atomic coordinates of the biomolecules may assist in capturing their diverse structural configurations. There are several theories of molecular recognition like lock-and-key model, induced fit and conformational selection that are hypothesized to explain the protein-ligand, protein-protein, protein-DNA, protein-RNA and RNA-ligand interactions participating in signaling, catalysis, gene regulation, and protein aggregation in health and disease. Nevertheless, very few studies have been reported on lectin-glycan binding reactions. Hence, we illustrate the basis of molecular recognition underlying lectin-glycan interactions by exploring the structural specificity of calreticulin family of chaperones, which specifically recognize and selectively bind to the monoglucosylated-N-glycan, among an existing pool of various high-mannose oligosaccharides in the endoplasmic reticulum. We performed molecular dynamics simulations to capture the complete conformational space represented by an ensemble of structures for distinct conformations. Structural evidences of distinct conformations of lectins in free and bound forms were characterized through free energy of binding, non-covalent interactions, protein-surface topography mapped by physicochemical properties like electrostatic and hydrophobic potentials to highlight and correlate the structural differences with the biomolecular behavior during binding. From this study, we noticed that both conformational selection and induced fit play a critical role in selective binding of the monoglucosylated-N-glycan and thus propose a mixed mechanism of binding in the calreticulin family of proteins.

## Introduction

Molecular recognition is a fundamental phenomenon behind many key biological processes, such as growth factor-receptor, antigen-antibody, receptor-ligand, repressor-DNA, RNA-ribosome, and lectin-glycan interactions [1,2]. Structural features and complementarity play decisive roles in realizing the requisite selectivity for the counterparts during interactions. The intrinsic play of biomolecules under binding interactions is governed by size, shape and intermolecular noncovalent interactions such as hydrogen bonding, hydrophobic forces, van der Waals forces, and electrostatic and CH/π interactions. Understanding them constitutes a cornerstone in chemical biology and drug discovery processes [3].

A paradigm of one such molecular recognition process is demonstrated by lectin chaperones participating in protein folding and quality control pathways in the endoplasmic reticulum (ER). Several molecular chaperones are actively engaged in streamlining the glycoprotein for either continued folding or degradation by distinguishing the proteins into natively folded, unfolded and terminally misfolded. The entire process functions with a high degree of precision on specifically recognizing different N-glycans covalently linked to polypeptides by different chaperones at different stages of the protein folding and quality control pathway [4–6]. During translocation into the ER lumen, nascent polypeptides are covalently modified by an enblock transfer of the tetradecasaccharide Glc_3_Man_9_GlcNAc_2_ (Glc: glucose, Man: mannose, GlcNAc: N-acetylglucosamine) to asparagine side chains in Asn-Xxx-Ser/Thr sequons. Subsequent trimming of sugar residues from the nonreducing end of N-glycans determines the fate of ER glycoproteins to ensure proper folding of glycoproteins while subjecting misfolded proteins to refolding or degradation [7].

The calreticulin family of proteins comprising the ER chaperone system includes calnexin (CNX), calreticulin (CRT), calmegin (CLMG) and calsperin (CALR3), which are well-characterized homologous lectin chaperones that transiently interact with newly synthesized glycoproteins. Calnexin is a type-I integral membrane protein, calreticulin is a soluble analog of calnexin within the ER lumen, and calmegin and calsperin are testis-specific homologs of calnexin and calreticulin, respectively [8–12]. Structurally CNX/CRT has three distinct domains. Their N-terminal region consists of a globular β-sandwich domain, while its P (proline-rich) domain has an extended curved arm connected to its N-terminal and C-terminal domains. The latter plays a role in binding to Ca^2+^ and its storage. The carbohydrate recognition domain (CRD) located within the N-terminal lectin binding domain recognizes Glc_1_Man_9_GlcNAc_2_ with high specificity; hence, its architecture is highly conserved in this family of chaperones [13].

Conformational transitions determine the relationship between the protein structure and function. The remarkable competence and structural specificity of molecular recognition can be explained by three models: (i) ‘Lock and Key’ model, which postulates shape complementarity in the binding site of the proteins to distinguish its ligands against others [14]; (ii) induced fit model posits conformational changes are induced in the molecule upon binding to its counterpart [15]; and (iii) conformational selection and population shift, in which an ensemble of conformations pre-exist and those complementary to ligands show preferential binding, thus signaling a functional shift among the conformers from one state toward a more select cohort of a stable complexes [16,17]. Since intermolecular recognition is governed by the shape and physicochemical properties and the local characteristics of the interacting partners’ surfaces, analyzing structural similarities among a family of proteins in terms of surface-specific patterns, fluctuations of functional groups, shape, distribution of charges and hydrophobicity will shed light on the functional dynamics of molecular recognition.

In this study, we explore the mechanism of molecular recognition in the calreticulin family of proteins by tracking the conformational changes in an ensemble of conformers under free and complexed forms, studying the protein surface topography to assess the electrostatic and hydrophobic properties of lectin CRD surfaces [18,19]. In addition to elucidating the structure-function relationship for the lectin-chaperone-glycan interaction, these studies highlight a hierarchy in the mode of this recognition wherein conformational selection precedes glycan-induced perturbation of the combining site residues for potentiation of the interaction.

## Results and discussion

In this study, a comprehensive analysis of the structural architecture and molecular specificity in the calreticulin family of proteins was performed to explain the specificity in recognizing its counterpart during lectin-glycan interactions. For this, a total of seven lectin chaperones of ER–calnexin and calreticulin–from various evolutionarily distinct species ranging from protozoans such as *Entamoeba histolytica* and *Trypanosoma cruzi* to mammals such as humans and dogs were used. Even the germ cell-specific variants of calnexin and calreticulin in humans, i.e., calmegin (CLMG) and calsperin (CALR3), were considered to associate structural features with the molecular recognition process. Calnexin and calmegin are membrane-bound proteins through the C-terminal transmembrane helix, whereas calreticulin and calsperin are soluble proteins [20]. Table 1 provides the details of the seven lectins in free and bound forms. The major glycan binding functionalities are rendered by their lectin domain, which specifically interacts with the monoglucosylated N-glycan [21–23]. Hence, we focused our analysis on the CRD region of these lectins.

**Table 1:** Seven molecular systems used in this study for molecular dynamics simulations and analysis.

Molecules	Name	Species obtained from	Abbreviation used
Lectin	Calnexin	*Canis lupus*	CNXC
	Calnexin	*Homo sapiens*	CNXH
	Calmegin	*Homo sapiens*	CLMG
	Calreticulin	*Homo sapiens*	CRTH
	Calsperin	*Homo sapiens*	CLSP
	Calreticulin	*Entamoeba histolytica*	CRTEh
	Calreticulin	*Trypanosoma cruzi*	CRTTc
Glycan	Monoglucosylated-N-glycan	-	CMONOG

### Sequence and structural analysis

**Fig 1:**
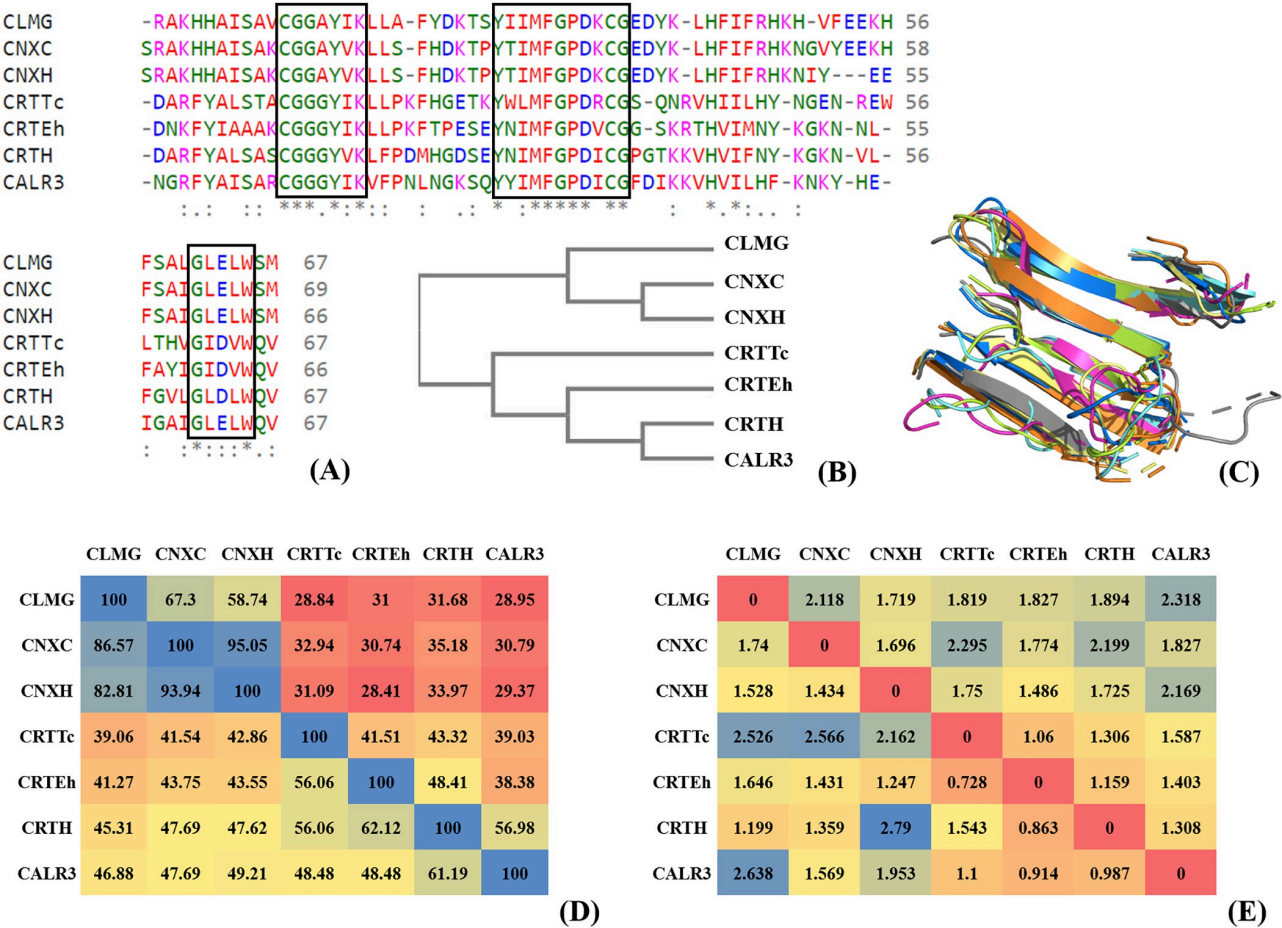
Sequence and structure alignment of carbohydrate recognition domain (CRD) residues of the seven calreticulin family of proteins. (A) Multiple sequence alignment of calnexin in dog (CNXC), human (CNXH), its homolog in testis—calmegin (CLMG), calreticulin in human (CRTH), *Entamoeba histolytica* (CRTEh), *Trypanosoma cruzi* (CRTTc) and its homolog in testis—calsperin (CALR3); (B) Phylogenetic tree showing the relationship between the calnexin and calreticulin and its homologs; (C) Superposition of the CRD region of proteins; (D) Heatmap showing the similarity of the aminoacids sequence–upper diagonal represent the whole sequence and lower diagonal for CRD residues; and (E) Heatmap of the root mean square deviation of the protein structures–upper diagonal represent structural deviation in whole proteins and lower diagonal for the CRD region only.

First, we examined sequence variation in the entire protein as well as the carbohydrate recognition domain (CRD) of calnexin and calreticulin in humans and protozoan parasites and their homologs in human male germ cells. Multiple sequence alignment of the whole sequences (S1 Fig) and the CRD region (Fig 1A) resulted in sequence similarities ranging between 28.41% to 67.3% and 39.06% to 93.94%, respectively, which indicates that the amino acid sequence of the CRD region is more conserved relative to the entire protein. A phylogenetic tree generated based on their complete sequences and their CRD region showed similar clustering of the proteins (Fig 1B). We noted that calnexin (CNXC and CNXH) and calmegin (CLMG) were grouped in a single cluster and that calreticulin of humans (CRTH) and protozoan parasites (CRTEh and CRTTc) and calsperin (CALR3) formed a separate cluster (Fig 1B). A heatmap of sequence similarities is shown in Fig 1D. The values in the upper diagonal represent similarities among whole protein sequences, and the lower diagonal represents similarities in the sequences of the CRD region. Additionally, structural superposition of the CRD region of the lectin chaperones was performed, and root mean square deviation (RMSD) were computed as a measure to study their structural similarities (Fig 1C) and represented them as a heatmap (Fig 1E). RMSD for whole proteins ranged from 1.06 (between calreticulins of protozoan parasites) to 2.318 (between calmegin and calsperin) and for CRD regions it varied from as low as 0.728 (between CRTEh and CRTTc) to as high as 2.79 (between CRTH and CNXH). Furthermore, sequences and structurally conserved residues were identified among the lectin chaperones and are presented in Table 2. This indicates that similarities were high among calnexin (CNXC, CNXH and CLMG) and calreticulin group of lectins (CRTH, CALR3, CRTEh and CRTTc), and larger differences were observed between calnexin and calreticulin at residue positions, viz. RP1, RP2 and RP13. This further intrigued us to investigate the structural modules in calnexin and calreticulin proteins and their association with the recognition of monoglucosylated N-glycan. Three common motifs–CGGXYXK-, -YXXMFGPDXCG- and–GXXXW- were located based on the sequence and structural alignment of the CRD regions (Fig 1A).

**Table 2:** Sequence and structurally conserved residues in the calreticulin family of proteins.

Residue Position	Secondary structure	Calnexin and its homologue			Calreticulin and its homologue			
		CNXC	CNXH	CLMG	CRTH	CALR3	CRTEh	CRTTc
RP1	β-strand 1/ Loop 1	K128	K127	K118	R73	R73	K64	R72
RP2	β-strand 1/Loop 1	H129	H128	H119	F74	F74	F65	F73
RP3	β-strand 2	C161	C160	C151	C105	C105	C97	C105
RP4	β-strand 2	Y165	Y164	Y155	Y109	Y109	Y101	Y109
RP5	β-strand 2	K167	K166	K157	K111	K111	K103	K111
RP6	β-strand 3	Y186	Y185	Y176	Y128	Y128	Y122	Y127
RP7	β-strand 3	M189	M188	M179	M131	M131	M125	M130
RP8	β-strand 3	D193	D192	D183	D135	D135	D129	D134
RP9	β-strand 3	C195	C194	C185	C137	C137	C131	C136
RP10	β-strand 4	K200	K199	K190	K143	K143	R136	R141
RP11	β-strand 4	H202	H201	H192	H145	H145	H138	H143
RP12	β-strand 4	I204	I203	I194	I147	I147	I140	I145
RP13	β-strand 5	E426	E425	E414	D317	E303	D312	D318
RP14	β-strand 5	W428	W427	W416	W319	W305	W314	W320

### Structural architecture of the carbohydrate recognition domain (CRD)

**Fig 2:**
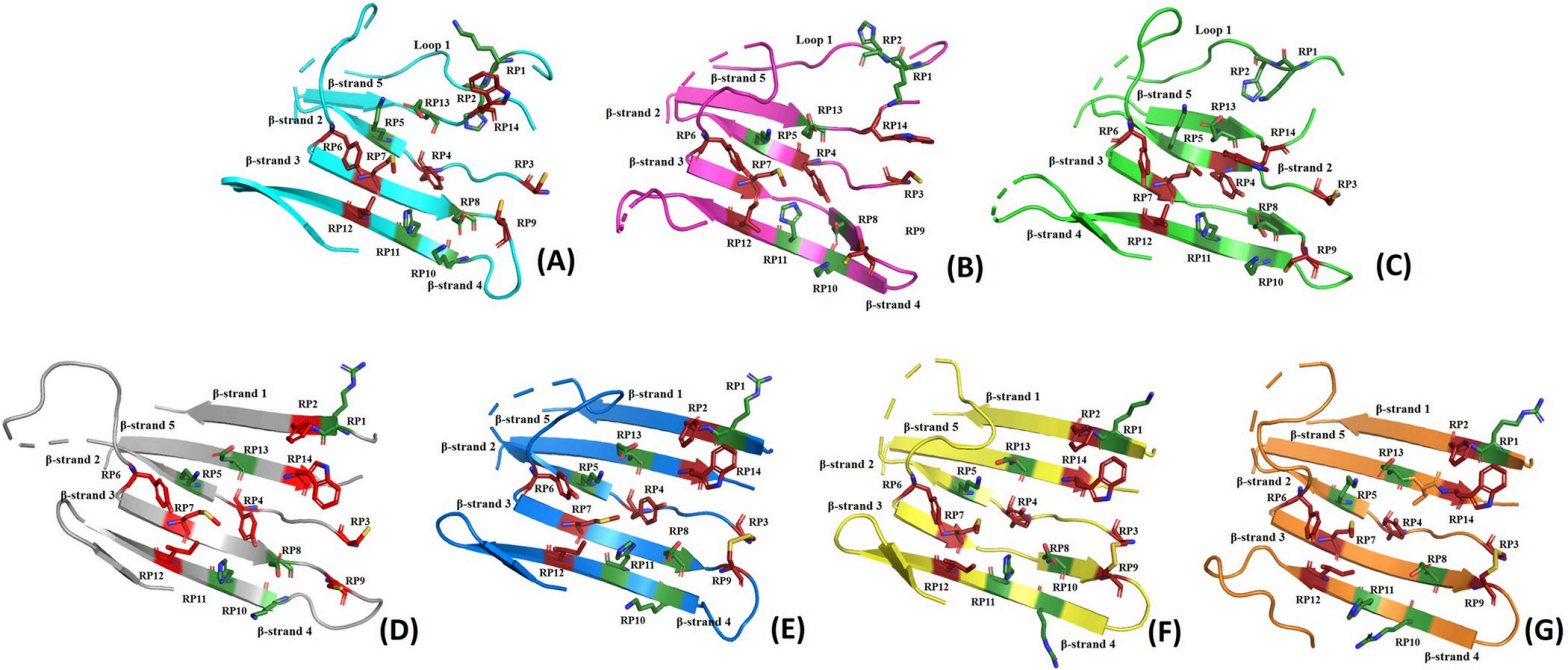
CRDs of the seven lectins showing the key conserved amino acid residues on the β strands. (A) Calnexin of *Canis lupus* (dog); (B) Calnexin of Humans; (C) Calmegin from Humans; (D) Calreticulin from Humans; (E) Calsperin from Humans; (F) Calreticulin from *Entamoeba histolytica*; and (G) Calreticulin from *Trypanosoma cruzi*. Polar amino acids are shown in green and non-polar amino acids in red.

Upon investigating the CRD, we found that the structural organization among calnexin and calreticulin proteins is highly conserved. All seven lectins had the same β-sheet architecture comprising four beta strands (β-strands 2–5) connected by loops and turns. Fig 2 shows the superposition of the CRD region and the key residues that are positionally and physicochemically conserved. The residue positions on different β-strands are marked as RP following the order of their occurrence in the sequence. A total of 14 residues were found to be conserved, of which the aromatic residues–Tyr and Trp residues (Y109, Y128 and W319) with respect to CRTH are at positions RP4, RP6 and RP14 (see Table 2), with implications in carbohydrate-aromatic interactions, were well conserved [24–26]. Findings of structural and functional similarities among the lectins with respect to these residues will be highlighted in the succeeding sections.

### Metastable conformations for functional analysis

For this study, lectin-glycan complexes were generated for all the seven lectins by docking monoglucosylated-N-glycan in their CRD and rendered for MD simulations for 200ns. S2 Fig illustrate the constructed lectin-glycan complex for CNXC, which was used for production simulations. Functional insights into the biomolecular complexes can be gleaned from molecular dynamics simulations. We performed classic MD simulations at the atomic level for all seven lectins and monoglucosylated N-glycans in free and complex forms. Considering that the whole trajectory for analysis may provide averaged approximations as they involve conformational transient forms, we followed a hierarchical sampling methodology to obtain metastable conformations existing in free and bound forms [27]. For this study, stable part of the trajectory was considered appropriate to sample conformations in deep energy wells to obtain biologically meaningful conformational transitions which govern induced fit or conformational selection that take place between shallow metastable states [28,29]. Subsequent to MD simulations, the trajectory was subjected to four variants of principal component analysis (all atoms-PCA and dihedral PCA), computed free energy landscapes for visualizing the conformational subspace, and geometrical separation of the clusters of conformations was facilitated via k-means clustering. S3 Fig shows the free energy landscapes of CNXC in free and complexed forms after subjecting to different variants of PCA and geometrical separation of conformational subspace for free (S3D Fig) and bound (S3I Fig) forms of CNXC. Table 3 shows the population size of different metastable conformers of molecular chaperone lectins in free and complexed forms. We observed that multiple conformers existed for the lectins in free form, but when bound to glycans, the number of conformers decreased in CLMG, CALR3, CRTEh and CRTTc, whereas calnexin and calreticulin of mammals (CNXC, CNXH and CRTH) exhibited multiple conformers before and after binding to the glycan. This can be explained by multifunctional roles of CNX and CRT, which allow them to associate with incompletely folded or conformationally trapped forms of glycoproteins through both glycan and protein binding interactions [30–33]. The restricted number of conformers of calreticulin in protozoan parasites (CRTEh and CRTTc) might be due to their interactions with a limited spectrum of proteins, such as human C1q in *Entamoeba* and *Trypanosoma* cruzi [34–36]. Sub-trajectories constituting metastable conformations of free and bound forms of lectins were considered for further analysis.

**Table 3:** Populations size of the metastable conformations of lectins in free and bound state obtained from hierarchical sampling methodology.

Lectin	State of the molecule	Cluster 1	Cluster 2	Cluster 3	Cluster 4
CNXC	Free	1727	5746	6353	-
	Bound	1303	980	2656	-
CNXH	Free	4753	3060	3411	-
	Bound	922	1573	3890	-
CLMG	Free	20890	-	-	-
	Bound	4155	-	-	-
CRTH	Free	1035	2348	1740	2872
	Bound	661	3485	922	1407
CALR3	Free	8742	-	-	-
	Bound	3625	-	-	-
CRTEh	Free	2369	3040	4022	
	Bound	6627	-	-	-
CRTTc	Free	2659	7685	-	-
	Bound	2230	-	-	-

### Trajectory analysis for structural stability

Structural changes during MD simulations were analyzed in all the free and bound forms through RMSD and radius of gyration. Root mean square deviation (RMSD) was computed to analyze the convergence in the 200 ns trajectories and the sub-trajectories with metastable conformations. The average RMSDs along with the standard deviations are provided in S1 Table. We observed low average RMSDs and standard deviations indicating that structural features are stabilized in the trajectories. S4–S7 Figs shows the RMSDs plotted as a function of time for both calnexin and calreticulin group of lectins in free and complexed forms. These plots show the occurrence of metastable conformations in different sub-trajectories obtained from the whole 200 ns trajectory. Furthermore, the sub-trajectories showed decreased standard deviation as compared to 200 ns trajectories (S1 Table), which confirms that the metastable conformations sharing similar conformational changes were binned into respective sub-trajectories following the hierarchical sampling approach [27].

Similarly, radius of gyration (Rg) was computed for the entire 200 ns trajectories as well as the sub-trajectories constituting metastable conformations for all the free and bound forms of the lectins (S8–S11 Figs). Radius of gyration describes the overall spread of the molecule indicating the compactness. Upon computing Rg values as a function of time, we found that free forms had relatively low Rg values than the complexed forms indicating that molecular packing in them is loosened after binding to the monoglucosylated-N-glycan (see S2 Table).

### Binding energies of different bound conformers

Selectivity and binding interactions determine the relative affinity of the underlying molecular recognition process. Hence, we calculated the relative binding energies of metastable conformers by ensemble averaging of 500 conformations sampled randomly following the molecular mechanics Poisson Boltzmann surface area (MMPBSA) approach as described [37]. The various components of binding energy, including electrostatic energy, van der Waals energy and solvation energy separated into polar and apolar regions, were computed and compared. Table 4 provides the relative binding energies and their components for all the metastable conformations of the lectin-glycan complexes. Considering the binding energy as the criterion to select the stable conformation, we noted that Clus3 of CNXC (-135.213 ± 10.481 kJ/mol), Clus1 and Clus3 of CNXH (-134.921 ± 2.500 kJ/mol and -134.134 ± 1.517 kJ/mol, respectively), Clus1 of CLMG (-105.570 ± 1.975 kJ/mol), Clus4 of CRTH (-27.620 ± 1.444 kJ/mol), Clus1 of CALR3 (-120.378 ± 1.417 kJ/mol), Clus1 of CRTEh (-51.304 ± 12.159 kJ/mol) and Clus1 of CRTTc (0.002 ± 4.261 kJ/mol) exhibited least binding energies, indicating high binding affinities, when compared to the other conformers of the lectin-glycan complexes. The sum of electrostatic and solvation energies suggests that most complexations are driven by van der waals energies, however, for CRTH the difference between them is large indicating a role of water mediated interactions for the stabilization. Hence, analysis of the water mediated hydrogen bonds revealed their existence between all the lectin-glycan complexes (S1 Data). However, they do not explain the observed differences between the electrostatic and solvation energies for CRTH. Furthermore, we investigated the differences in the conformational changes among the various clusters and their molecular interaction fields to highlight the differential architecture of the CRD among the different lectins and their contribution to binding affinities.

**Table 4:** mmPBSA calculations of various components of non-covalent binding energies for an ensemble of conformations in calnexin, calreticulin and their homologues bound to monoglucosylated-N-glycan.

Lectins	Clusters of different conformers	van der Waal energy (kJ/mol)	Electrostatic energy (kJ/mol)	Polar solvation energy (kJ/mol)	SASA energy (kJ/mol)	Binding energy (kJ/mol)
CNXC	Clus1	-145.359 ± 0.966	-260.533 ± 2.046	310.429 ± 4.794	-26.206 ± 0.124	-121.449 ± 4.385
	Clus2	-140.454 ±1.044	-252.649 ±2.018	331.387 ± 10.656	-26.396 ± 0.116	-87.762 ± 10.290
	Clus3	-178.287 ± 0.851	-267.510 ± 1.778	339.657 ± 10.859	-28.912 ± 0.080	-135.213 ± 10.481
CNXH	Clus1	-188.143 ± 1.083	-216.047 ± 1.793	297.579 ± 3.091	-28.351 ± 0.137	-134.921 ± 2.500
	Clus2	-178.378 ± 1.174	-265.122 ± 2.357	346.403 ± 2.551	-28.262 ± 0.110	-125.248 ± 1.347
	Clus3	-207.842 ± 1.137	-262.745 ± 2.641	370.699 ± 2.917	-34.145 ± 0.103	-134.134 ± 1.517
CLMG	Clus1	-178.890 ± 0.935	-165.817 ± 1.906	265.147 ± 2.884	-26.051 ± 0.107	-105.570 ± 1.975
CRTH	Clus1	-113.522 ± 1.153	-76.066 ± 1.310	203.746 ± 1.968	-21.016 ± 0.149	-7.024 ± 1.520
	Clus2	-138.157 ± 1.855	-84.431 ± 1.683	263.877 ± 12.522	-22.759 ± 0.225	19.134 ± 13.178
	Clus3	-105.104 ± 1.394	-68.733 ± 1.561	217.974 ± 2.437	-19.038 ± 0.208	25.050 ± 1.721
	Clus4	-115.647 ± 1.169	-84.748 ± 0.940	193.621 ± 1.450	-20.954 ± 0.189	-27.620 ± 1.444
CALR3	Clus1	-72.629 ± 0.786	-159.112 ± 1.272	125.707 ± 1.589	-14.340 ± 0.110	-120.378 ± 1.417
CRTEh	Clus1	-80.786 ± 0.649	-150.861 ± 1.101	198.421 ± 12.349	-18.232 ± 0.084	-51.304 ± 12.159
CRTTc	Clus1	-161.367 ± 0.725	-141.343 ± 0.850	328.874 ± 4.368	-26.015 ± 0.077	0.002 ± 4.261

### Relative analysis of the carbohydrate recognition domain

**Fig 3:**
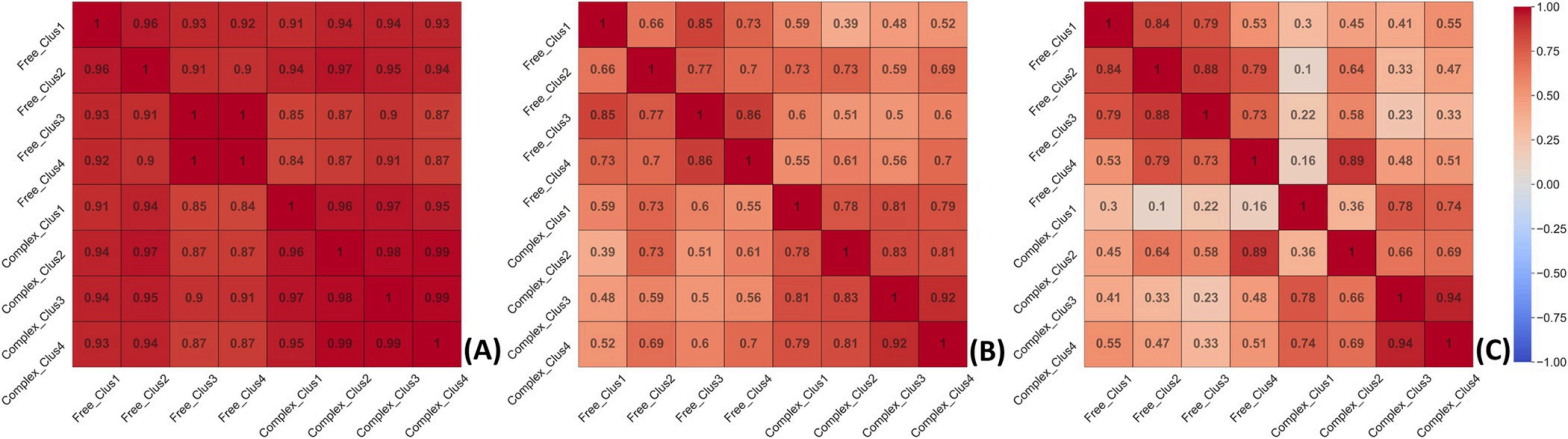
Heatmaps showing the relative similarities among various clusters of free and bound forms of CRTH: (A) SASA; (B) RMSF of CRD and (C) RMSF of conserved residues.

Subsequently, we analyzed the dynamics of CRD as biomolecular recognition induces protein conformational changes in the interacting partners [38]. Previously, several studies have applied SASA to detect protein structural changes, by assessing the local environment and the interface of the protein in its complex state [39–41]. In this study, we explored the changes in solvent accessible surface area (SASA) by computing Pearson correlation coefficients to measure the similarities in structural rearrangements between the conformers of free and bound forms that occurred due to glycan interactions. Color-coded heatmaps represent the similarity in the CRD between the different clusters (Figs 3 and S12–S17). We noticed significant similarities in SASA of CRDs—as high as 85–99% between the conformers of free and bound forms indicating small-scale conformational changes upon complexation in the CRD of the seven lectins (see Fig 3A). We then investigated the similarities in the dynamic fluctuations of the CRD in free and bound forms of the lectins in the form of RMSFs of the aminoacids. Pearson’s correlation coefficient based on RMSFs was found to be between 0.39–0.73 for CRTH (Fig 3B), 0.53–0.83 for CNXC, 0.6–0.77 for CNXH, 0.6 between the free and bound clusters of CLMG, 0.85 for CALR3, 0.68–0.94 for CRTEh, and 0.15–0.74 for CRTTc for the residues of CRDs (S12B–S17B Figs). This indicated a decreasing trend in similarities in RMSFs of CRD relative to SASA which prompted us to investigate the RMSFs of the fourteen key residues (Table 2). Fig 3C represents the correlogram based on the RMSFs computed for the fourteen conserved residues in CRTH to represent the level of synchrony in residue motions. We noted variable correlation coefficients ranging from 0.1 to 0.89 in CRTH (Fig 3C), 0.06–0.73 in CNXC, -0.018–0.67 in CNXH, 0.31 in CLMG, 0.67 in CALR3, 0.56–0.96 in CRTEh and 0.19–0.65 in CRTTc (S12C–S17C Figs). The solvent accessible surface area (SASA) between the free and bound forms are highly conserved, while the fluctuation of amino acids in the CRD (RMSFs) specifically the fourteen key residues increased. This indicates that although the CRD architecture remained stable, the movements in the amino acids were influenced by the binding of glycan. However, we noticed high similarities among the various conformers of the bound forms, indicating that under favorable conditions, significant fluctuations in the residues of the CRD may lead to conformational changes resulting in population shift among the conformers toward the most favorable conformation. Hence, we analyzed the noncovalent interactions between the glycan and the lectin and consequent changes in the protein surface topography by mapping physicochemical properties of their CRDs.

### Relative analysis of molecular interaction fields (MIFs)

**Fig 4:**
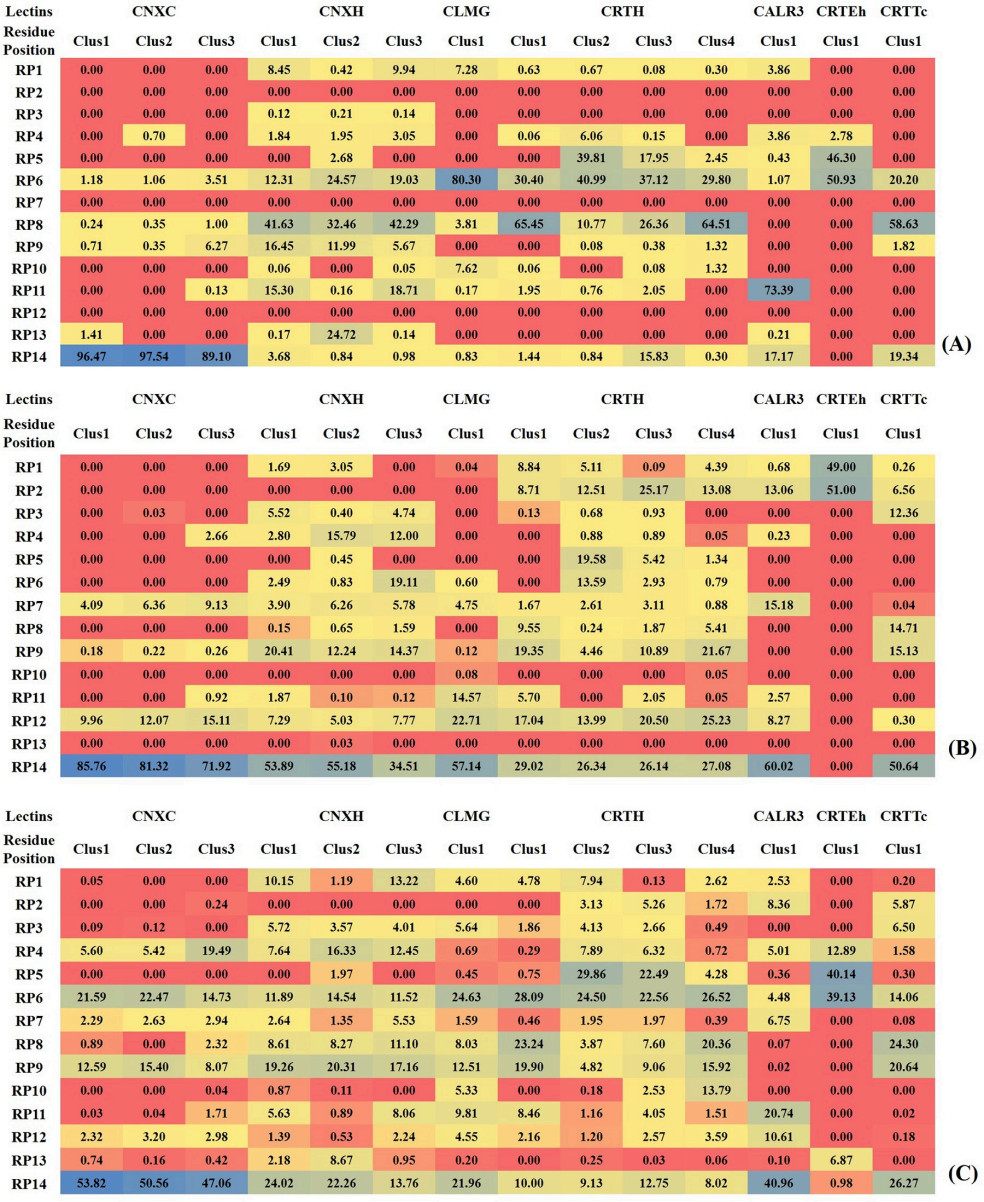
Heatmaps showing the residue position-wise noncovalent bond interactions in clusters of lectins: (A) hydrogen bonding of various conformers with monoglucosylated N-glycans; (B) hydrophobic interactions; and (C) van der Waals contacts.

**Fig 5:**
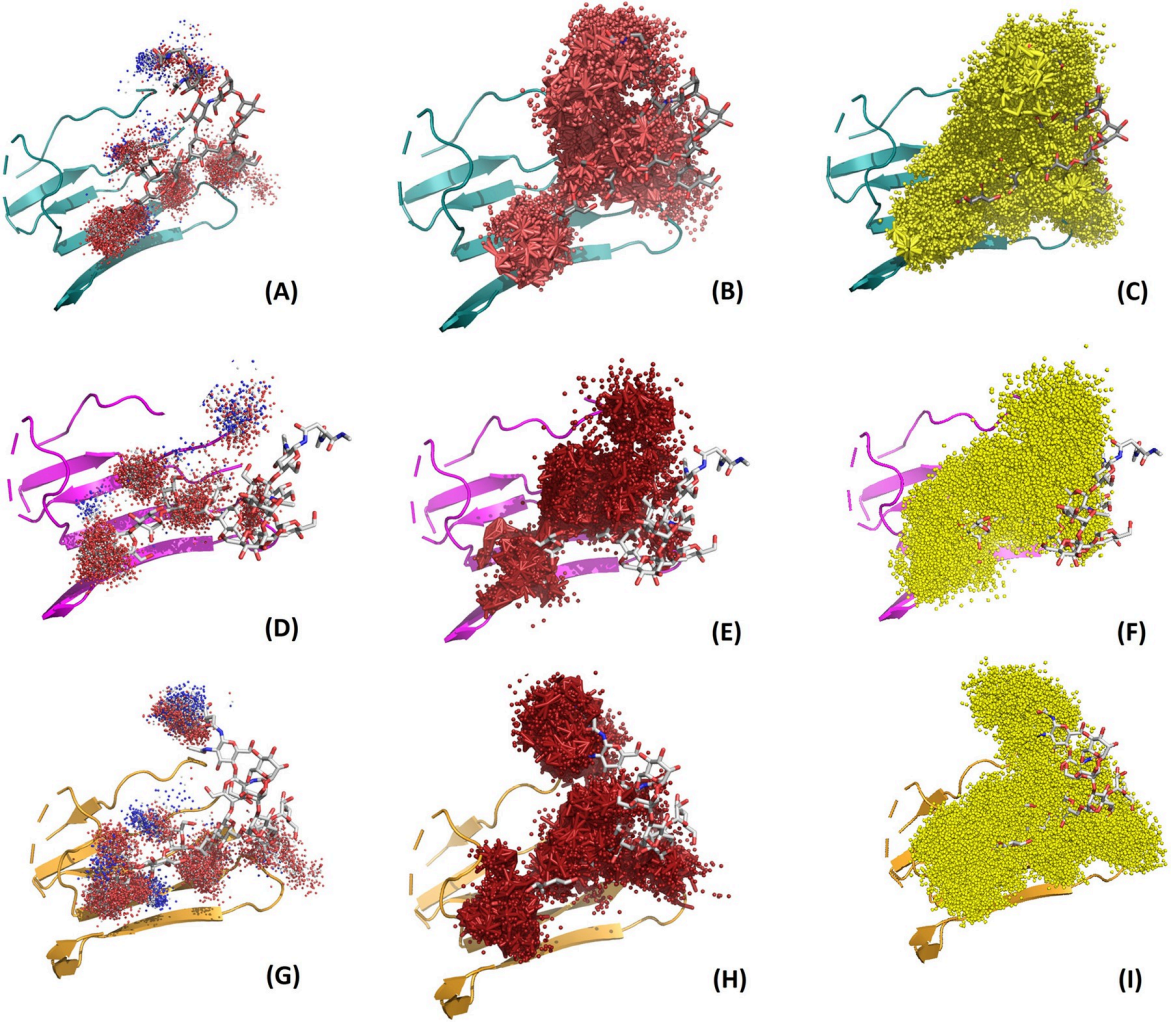
Molecular interaction fields of CNXH upon binding to monoglucosylated-N-glycan computed for the three metastable clusters: (i) Complex_Clus1 (A) Hydrogen bonding interactions; (B) Hydrophobic interactions and (C) van der Waals interactions; (ii) Complex_Clus2 (A) Hydrogen bonding interactions; (B) Hydrophobic interactions and (C) van der Waals interactions; and (iii) Complex_Clus3 (A) Hydrogen bonding interactions; (b) Hydrophobic interactions and (C) van der Waals interactions.

The physicochemical environment of the CRD can be encoded by molecular interaction fields that capture the functional behavior of the key residues of the protein. Here, relative similarities in the chemical environments were demonstrated based on the noncovalent bond interactions between these lectins and monoglucosylated N-glycans. Since similarities based on the number of atoms might not reflect differences in the conformers of the same molecule, we computed the similarities between the CRD regions by exploring interactions such as hydrogen bonds and hydrophobic and van der Waals interactions using the trajectories constituting metastable conformations (see S2 Data for the noncovalent bond interactions observed in different clusters of lectin-glycan complexes). The binding interactions with the conserved residues mapped on the CRD region of the seven lectins (Table 2) were used to visualize the similarities as heatmaps. We observed that Tyr and Trp residues at RP6 and RP14 were involved in hydrogen bonding interactions in all the conformers of all the lectins, which had implications for aromatic interactions with glycan [24,25]. Fig 4(A) shows the percentage occurrence of hydrogen bonds between the conserved residues of the CRD of lectins and the hydroxyl groups of the monoglucosylated N-glycan. Likewise, analysis of hydrophobic interactions among different clusters highlighted the involvement of key conserved residues, viz. Met, Cys, Ile and Trp at positions RP7, RP9, RP12 and RP14, respectively (Fig 4B). Furthermore, residues at positions RP4, RP6, RP7, RP9, RP11, RP12 and RP14 were in close contact with the glycan through van der Waals interactions. Interestingly, these included mostly aromatic hydrophobic residues (Fig 4C). Thus, it is apparent that aromatic and hydrophobic amino acids, i.e., Trp and Tyr are markedly preferred in the CRD region and are involved in the molecular recognition process [42]. Moreover, exploring the molecular interaction fields of the lectin-glycan interactions, we noted that Tyr (RP6) formed bonds with the terminal glucose residue in the A-arm and Trp (RP14) was in close proximity to the mannose residues of the A- and B-arms. Molecular interaction fields for the other seven lectins are shown in Figs 5 and S18 –S23). Furthermore, we have checked whether there are significant differences in the MIFs between the different metastable clusters of the same lectin-glycan complex and computed separately. But we did not notice prominent differences in the MIF clouds other than displacement in the positions, which is due to the flexible glycan and loop region of the lectin. Fig 5 shows the MIFs generated for the three clusters of CNXH, which showed least distinction between the clusters.

### Relative protein surface topography (PST) of CRD

**Fig 6:**
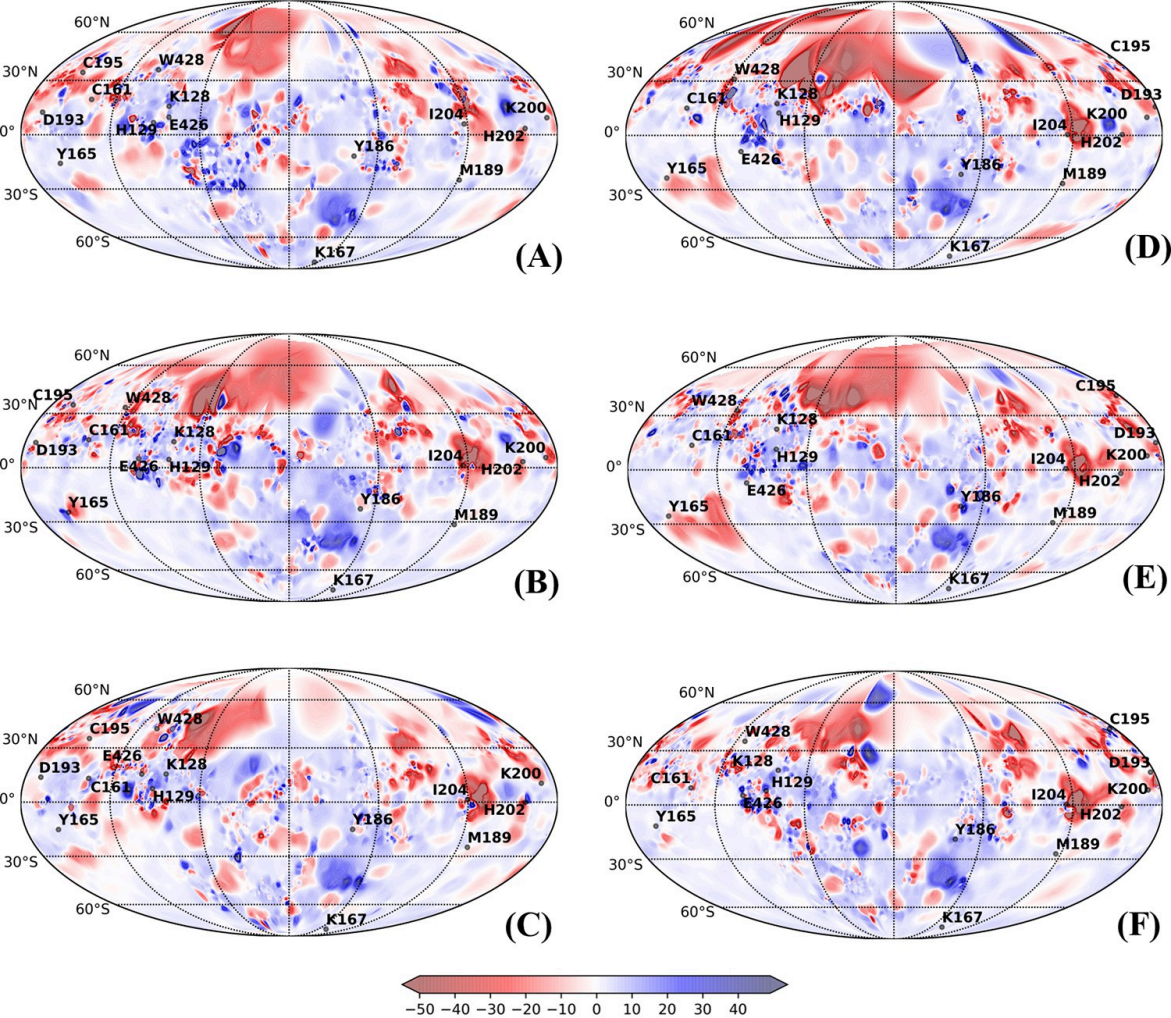
Protein surface topography representing molecular electrostatic potentials (MEP) localized to the CRD region with fourteen key residues were mapped onto 2D spherical projections for visual comparison of free and bound forms of Calnexin in *Canis lupus* (CNXC): (A) Free_Clus1; (B) Free_Clus2; (C) Free_Clus3; (D) Complex_Clus1; (E) Complex_Clus2; and (F) Complex_Clus1.

**Fig 7:**
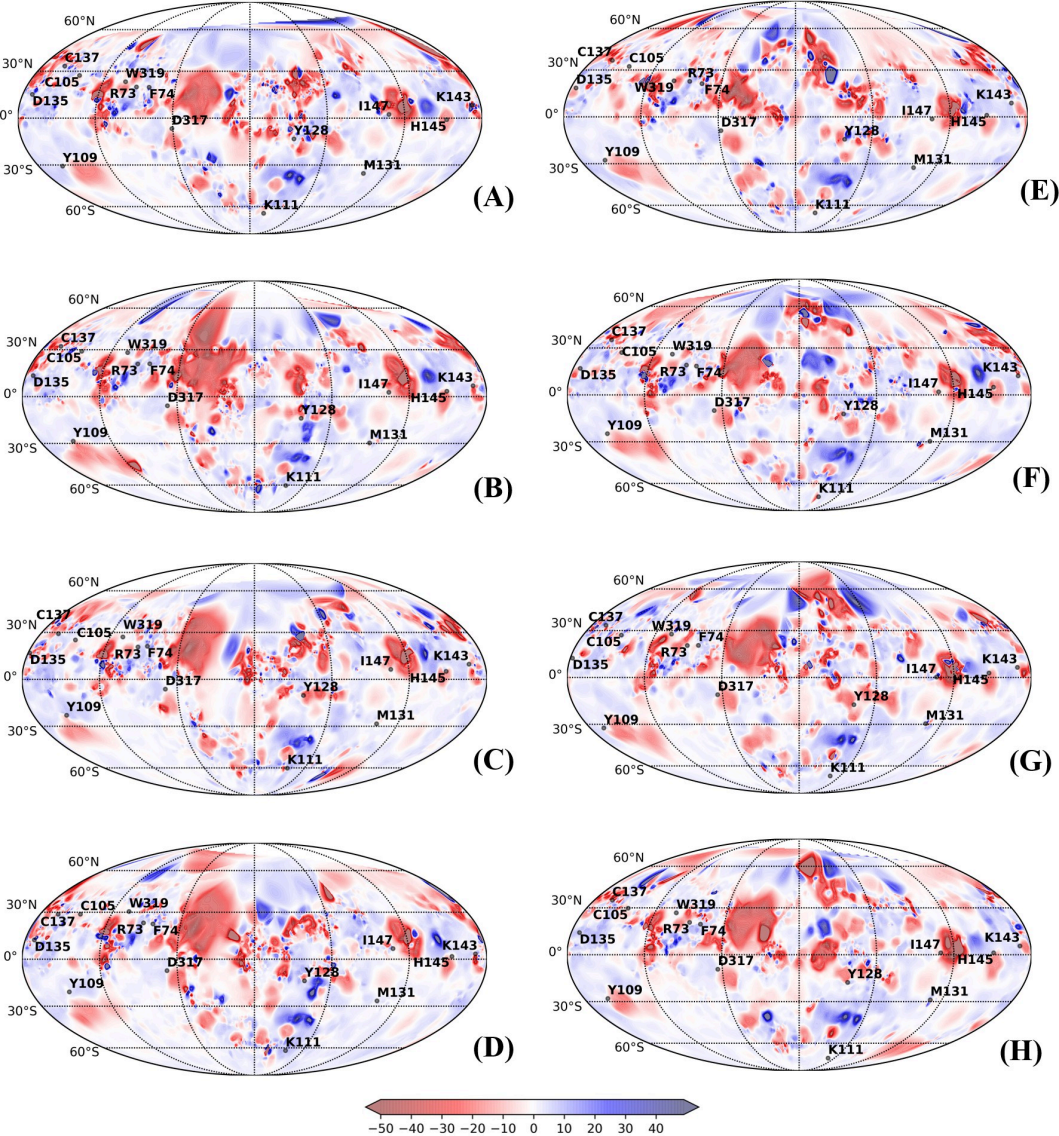
Protein surface topography representing molecular electrostatic potentials (MEP) localized to the CRD region with fourteen key residues were mapped onto 2D spherical projections for visual comparison of free and bound forms of Calreticulin in Humans (CRTH): (A) Free_Clus1; (B) Free_Clus2; (C) Free_Clus3; (D) Free_Clus4; (E) Complex_Clus1; (F) Complex_Clus2; (G) Complex_Clus3 and (H) Complex_Clus4.

PST is well suited for visualization of conformational transitions and mapping physiochemical properties on the surface of multistate proteins [19,43,44]. PSTs have proven to be an efficient tool to visualize conformational transitions and molecular properties of small G-proteins, mapping of caspase-1 intersubunit interface and complementarity in conotoxins-acetylcholine protein complex [19]. PSTs were also exploited to increase the binding affinity of Tk-hefu peptides to potassium channel. Mapping of electrostatic potential and hydrophobic potentials assisted in enhancing selective activity of potassium channel blocker and to design and optimize specific ligands [43]. Yet, in another study, structural comparison, mapping of hydropathic and dynamic properties of α-like toxins of scorpion venom on the molecular surface revealed the molecular determinants underlying selective toxicity [44]. Here, we applied PST method to compare the conformational transitions and changes in the physiochemical properties between free and bound lectins and relate them to their biological function We mapped the electrostatic and hydrophobic potentials onto the spherical projection maps obtained for the representative structures from each of the subtrajectories of different conformers. Figs 6 and 7 show the structural dynamics and molecular electrostatic potentials (MEPs) of calnexin (CNXC) and calreticulin (CRTH) mapped on 2D spherical projections. PSTs for other lectins are provided S24–S28 Figs showing MEP between the free and bound conformation. Qualitative analysis of the maps showed no drastic change in the spatial distribution of the MEP around the conserved residues of the free and bound states of the lectins, which emphasizes that the physiochemical properties of the surface remained mostly unaltered. We noticed that MEP islands of the bound forms shared higher resemblances with the free forms, however a slight drift in islands was noted, which is obvious due to binding dynamics. These shared similarities ascertain prior existence of conformations of lectins in free form that interact and can bind with glycan and higher similarities among bound forms confirm occurrence of populations shift towards the favorable conformation. The amino acid residues at positions RP4- RP7 and RP10 –RP12 on β-strands 2, 3 and 4 were present in positive electrostatic potential with low electron density repelling the protons (shades of blue), whereas other residues are positioned in negative electrostatic potential that attract protons by the concentrated electron density (shades of red).

**Fig 8:**
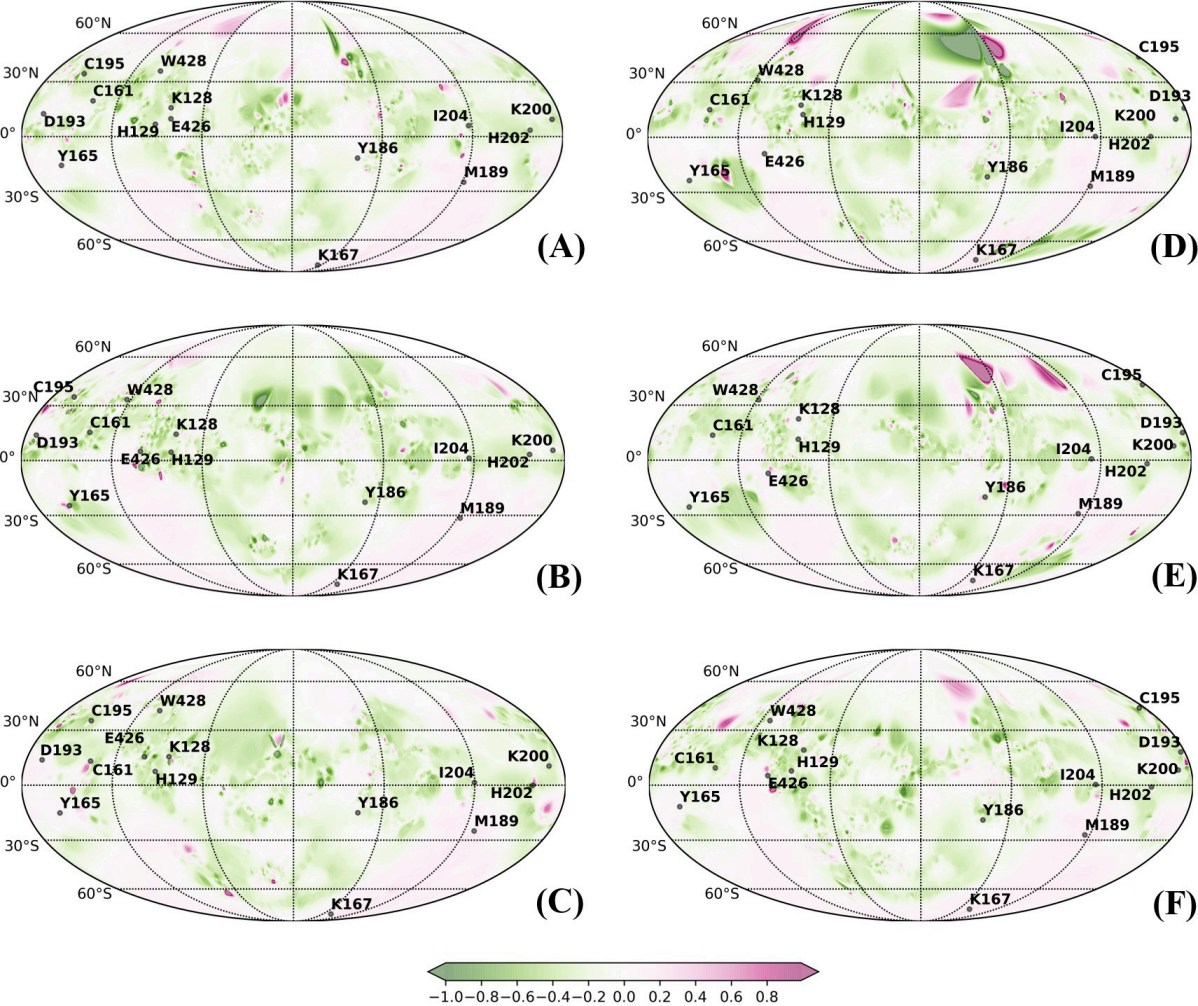
Protein surface topography representing molecular hydrophobic potentials (MHP) localized to the CRD region with fourteen key residues were mapped onto 2D spherical projections for visual comparison of free and bound forms of Calnexin in *Canis lupus* (CNXC): (A) Free_Clus1; (B) Free_Clus2; (C) Free_Clus3; (D) Complex_Clus1; (E) Complex_Clus2; and (F) Complex_Clus1.

**Fig 9:**
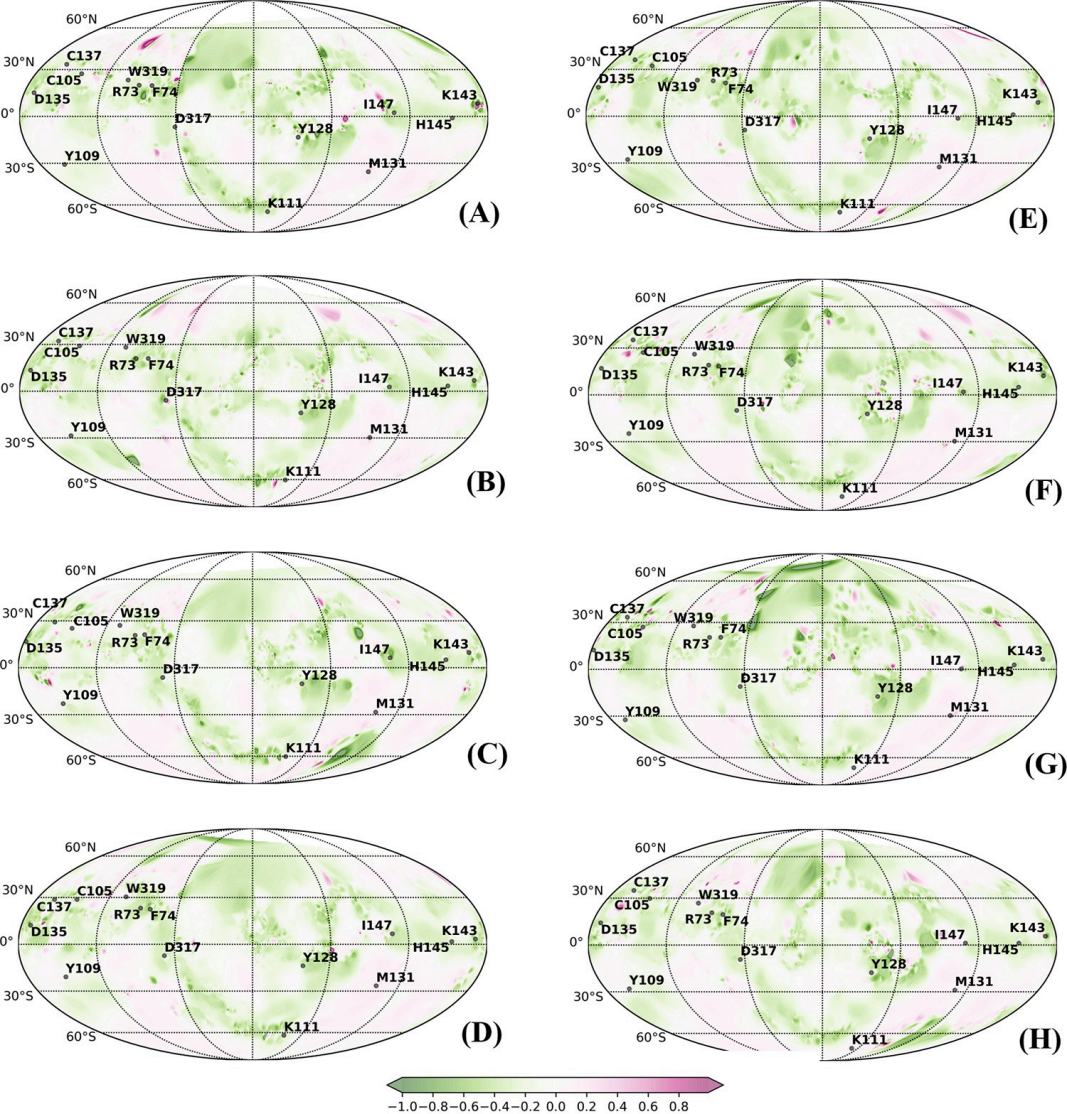
Protein surface topography representing molecular electrostatic potentials localized to the CRD region with fourteen key residues were mapped onto 2D spherical projections for visual comparison of free and bound forms of Calreticulin in Humans (CRTH): (A) Free_Clus1; (B) Free_Clus2; (C) Free_Clus3; (D) Free_Clus4; (E) Complex_Clus1; (F) Complex_Clus2; (G) Complex_Clus3 and (H) Complex_Clus4.

Likewise, analyzing the CRD surface by mapping the molecular hydrophobic potential showed that the CRD region is predominantly hydrophilic in nature in all lectins (Figs 8, 9 and S29–S33). Analyzing the 2D spherical projection maps further indicated that the spatial distribution of molecular hydrophobic potentials (MHP) is not very different between the free and bound states or among the different lectins. All the conserved residues are predominantly located in hydrophilic zones, and although slight dynamic changes occurred due to glycan interactions, the MHP islands were not altered.

As PSTs assist in visualizing the dynamic changes in the structural organization, we scrutinized the changes in the CRD region due to glycan binding based on the fourteen structurally and functionally conserved residues positioned in five β-strands (β1 –β5) (Table 2). Seven amino acids at positions RP4-RP7 and RP10-RP12 on β-strands 2, 3 and 4 were more rigid in their movement, while the other seven residues at positions RP1-RP3 and RP8, RP9, RP13 and RP14 on β-strands 1 and 5 were relatively dynamic in all the lectins of this family.

In summary, a comprehensive study was performed to answer the fundamental question of molecular binding by the calreticulin family of proteins as a paradigm. Conformational changes in calreticulin, calnexin and their homologs in humans and other distant species illustrated the relationship between their structural architecture and functional dynamics. High similarity in the protein sequence and architecture of CRD regions was observed as compared to the rest of the protein in them. Hence, we marked fourteen residues that were structurally conserved and relevant to their glycan binding. Since proteins can exist in an ensemble of conformers during free and bound states, metastable conformations in their ground state were identified through a hierarchical sampling approach. Calnexin and calreticulin in higher animals existed in multiple conformers than their homologs, i.e., calmegin, calsperin and calreticulin from protozoan parasites which, we attribute to the existence of multiple conformations in the lectins from the higher organisms due to their interactions with a multitude of glycoproteins. On the other hand, their homologs from lower level organisms have a very specific role of interacting with the glycans only limiting their conformational variation to one final form after binding. Ground states among the different molecular conformations were identified based on binding energies for the seven lectins. Furthermore, analysis of SASA and RMSFs on an ensemble of conformations confirmed structural stability in the CRD across the lectins, but residue fluctuations were noted in the fourteen key residues. A closer inspection of the molecular interaction fields of the CRD among the various bound conformers of all seven lectins demonstrated the apparent role of the conserved aromatic and hydrophobic amino acids, i.e., Tyr and Trp at RP6 and RP14 for specific binding to monoglucosylated N-glycans. Additionally, we investigated the significance of physicochemical properties by comparing the protein landscapes mapped by electrostatic and hydrophobic potentials on the surface. Qualitative analysis of the PSTs illustrated similar MEPs and MHPs between the free and bound forms in the seven lectins upon interactions with the glycan highlighting that the structural and physiochemical properties are preserved in the calreticulin family of proteins to drive conformation selection. However, fluctuations in the key conserved residues appear to enhance their binding. Based on these findings, we suggest that the calreticulin family of proteins while exist in a favorable molecular conformation in terms of structure and physicochemical properties to recognize the monoglucosylated N-glycan, further fluctuations in the conserved residues of the CRD promote efficient glycan binding to accomplish their functional role in the protein folding process.

This work was initially designed to determine whether conformational changes in lectins during glycan binding occur through conformational selection or induced fit mechanism. For this, we relied on molecular dynamics simulations to uncover the mechanism of biomolecular recognition and quantify the biomolecular interactions in a calreticulin family of proteins known to share similar structural architecture and binding specificities. Upon exploring the molecular subspace in free and bound states of the lectins, we witnessed multiple conformations with high similarities in structural dynamics of carbohydrate recognition domain and physicochemical properties revealing pre-existing conformations in lectins even before binding to the glycan. Subsequently, glycan interactions induced conformational changes in the CRD involving conserved residues further strengthening the binding process. In summary, a mixed mechanism involving conformational selection followed by induced-fit mechanism could provide a satisfactory explanation for complex biological recognition process.

While these studies provide insights into the binding mechanism of calreticulin family of proteins, there are some limitations of the study. As the three-dimensional structure for calnexin, calmegin and calsperin from humans were lacking, we had to rely on homology modeling to build the molecules. Also, obtaining the trajectory that captured the dynamics of glycan in the lectin-CRD during molecular dynamics simulations was quiet challenging, as CRDs exist as shallow depression on the surface of these lectins. Additionally, the high degree of flexibility in the high-mannose glycan was cumbersome. Consequently, identifying and tracking the path of conformational transition between the free and bound conformers remained elusive.

## Methodology

### (i) Obtaining protein sequences

Protein sequences for calnexin (*Canis lupus familiaris* (CNXC); UniProt ID: P24643), calnexin (*Homo sapiens* (CNXH); UniProt ID: P27824), calreticulin (*Homo sapiens* (CRTH); UniProt ID: P27797), calreticulin (*Entamoeba histolytica* (CRTEh); F2VNV2), calreticulin (*Trypanosoma cruzi* (CRTTc); Q4CPZ0), calsperin (*Homo sapiens* (CALR3); UniProt ID: Q96L12) and calmegin (*Homo sapiens* (CLMG); UniProt ID: O14967) were taken from the UniProt sequence database (https://www.uniprot.org/).

### (ii) 3D structures of lectins

X-ray diffraction structures for calnexin (CNXC; PDB ID: 1JHN), calreticulin (CRTH; PDB ID: 3POS), calreticulin from *E*. *histolytica* (CRTEh; 5HCA), and calreticulin from *T*. *cruzi* (CRTTc; 5HCF) were available in the RCSB protein databank (https://www.rcsb.org/) [45–47]. Since three-dimensional structures for calnexin (CNXH), calsperin (CALR3) and calmegin (CLMG) in *Homo sapiens* were not available, homology modeling was performed using CNXC and CRTH as templates using MODELLER 9v2 (https://salilab.org/modeler/) [48]. The 3D coordinates for the monoglucosylated N-glycan (Glc_1_Man_9_GlcNAc_2_-Asn; MonoG) structure (LINUCS ID: 13292) was obtained from SweetDB (http://www.glycosciences.de/sweetdb/) [49]. Furthermore, lectin-glycan complexes were prepared using PyMOL software (The PyMOL Molecular Graphics System, version 1.2r3pre, Schrödinger, LLC) [50]. PDBs of lectin molecules complexed with disaccharides (with terminal glucose) or tetra-saccharide were used to dock the monoglucosylated-N-glycan in the CRD of the lectins based on the orientation of the disaccharide or tetrasaccharide.

### (iii) Molecular dynamic simulations

All-atomistic molecular dynamics simulations were performed for the seven free lectin molecules, free monoglucosylated N-glycans and their respective complexes using GROMACS v5.1.1 [51]. Molecular systems were prepared using the OPLS-AA force field and TIP3P water model [52–54]. Initially, energy minimization in vacuum was performed for 1000 steps using the steepest descent algorithm followed by conjugate gradient minimization. Periodic boundary conditions were defined by adjusting the boundaries of the cubic box by 10 Å. Water was added to the unit box and neutralized using sodium and calcium ions to maintain overall charge neutrality. Energy minimization was performed for the solvated systems for 5000 steps until the maximum force applied was less than 1,000 kJ/mol/nm. Position-restrained MD simulations was performed for 500 ps by fixing the backbone atoms of the solute to allow the solvent to adapt to the molecule. Unrestrained MD simulations were performed by coupling to a temperature bath set to 300 K and a pressure bath at 1 atm using a Berendesen thermostat and Parinello-Rahman barostat, respectively. A leap-frog integrator was implemented with a time step of 2 fs. All bonds were constrained using LINCS algorithm. Electrostatic calculations were accounted for by the particle mesh Ewald (PME) method with a cutoff distance for Coulomb and van der Waals interactions maintained at 1.4 nm. Production simulations for eight free and seven bound molecular systems were run for 200 ns.

### (iv) Hierarchical sampling for metastable conformations

The MD trajectories were subjected to hierarchical sampling to obtain metastable conformations following the procedure described [27]. Four different variants of principal component analysis (PCA), free energy landscapes and k-means clustering algorithms were implemented to separate the conformations showing functionally concerted atomic motions. For this, (i) harmonic motions of all the atoms (cPCA) of the free or molecular complex were accounted for in level 1, (ii) dihedral PCA (dPCA_b_) was applied on the backbone atoms in level 2, (iii) dihedral torsion angles (phi (Φ), psi (Ψ), chi (χ) torsion angles) restricted to the carbohydrate recognition domain (dPCA_crd_) in lectins and glycan (phi (Φ), psi (Ψ) and omega (ω) torsion angles) were accounted for in level 3, and (iv) only the dihedral torsion angles of the glycan (dPCA_gly_) were accounted for lectin-glycan complexes in level 4. Free energy landscapes (FELs) were used to visualize the essential subspace and to bin the conformations exhibiting harmonic motions. K-means clustering was applied to enable geometric separation of the clusters constituting an ensemble of metastable conformers followed by generating subtrajectories for each conformer for further analysis.

### (v) Preliminary analysis of MD Trajectories

Sub-trajectories of various metastable conformers were analyzed by computing root mean square deviations (RMSD) for the backbone atoms of the molecular systems. Radius of gyration (Rg) was computed on the backbone atoms of the lectins for the whole trajectory as well as sub-trajectories to evaluate the structural stability. Residue-wise fluctuations of the CRD were calculated using the root mean square fluctuations (RMSF). The solvent accessible surface area for the CRD was calculated for both the free and bound forms of the lectins.

### (vi) Estimation of glycan binding affinity of various lectins

Binding energies of the lectin-glycan complexes were computed using the g_mmpbsa package developed [37]. The tool computes the binding energy by applying the mmPBSA method except the entropy term, which is the sum of the energy terms computed for the components of molecular mechanisms (van der Waals and electrostatic energy) and polar and nonpolar solvation energies. The sub-trajectories for different conformers of the lectins were used to compute the binding energies to determine their relative strength of interactions with the monoglucosylated N-glycan. A total of 500 conformers from each sub-trajectory were deposited randomly to compute the binding energies to avoid bias during relative comparison.

### (vii) Molecular interaction fields (MIFs)

A three-dimensional potential map describing the interactions between the calreticulin family of proteins and the monoglucosylated N-glycan was generated using BINANA (BINding ANAlyzer) (https://durrantlab.pitt.edu/binana/). The trajectory of metastable conformations of separated clusters was analyzed for binding interactions. The program identifies noncovalent bond interactions such as hydrogen bonds, hydrophobic interactions and van der Waals interactions. In-house scripts were used to collate the molecular interactions for the conformations in the subtrajectories to generate molecular interaction fields and for relative comparison between the conformers within and between the various clusters of lectins. Color-coded heatmaps were generated to visualize and compare the occurrence of noncovalent binding interactions observed with the key conserved residues of lectins.

### (viii) Electrostatic potential maps

Electrostatic potentials were mapped onto the protein surface using APBS software [55,56]. Calculations were performed on the three representative structures randomly taken from each subtrajectory of conformations from the clusters of free and bound lectins. The pdb2pqr program was used to assign charges to the proteins based on the atomic radii of the AMBER force field [57]. To compute the electrostatic potentials, a fine grid spacing of 0.25 Å was used. Solvent effects were accounted for using a 0.15 M 1:1 electrolyte and dielectric constants of 2.0 and 78.54 for the protein interior and the solvent water, respectively. APBS software computes the potential surface by solving the nonlinear PB equation in a grid-based approach. The electrostatic potentials were computed for every point in the grid space, and the values are exported in a. dx file. The electrostatic potentials were mapped onto the Connolly surface of the lectin-CRDs using the MSMS program [19]. The electrostatic potentials were then mapped onto the CRD surfaces through protein surface topology to compare the relative changes in the physicochemical properties due to glycan binding.

### (ix) Hydrophobic potential maps

The molecular lipophilicity potential (MLP) was calculated for the representative structures using pyMLP.py [58,59]. The Python script contains a library of atomic lipophilic potential values for every atom based on its chemical properties and calculates MLP in every point of the grid in the protein space.

$$MLP(r)=\sum_{i}f_{i}e^{\frac{-|r-r_{i}|}{2}}$$

where r is a point in the protein space, fi is the atomic lipophilic potential for atom i, and ri is the position of atom i.

pyMLP outputs a.dx file in which the header defines the grid origin, the grid step and the number of points on each axis. The MLPs were then mapped to the CRD of the lectins in different conformers to enable comparison between the conformers of free and bound forms as previously described for mapping electrostatic potentials.

### (x) Protein surface topography (PST)

Protein surface variability in terms of physiochemical properties was studied by computing the PST for all seven calreticulin family of proteins in free and bound forms following the procedure of [19]. Although the procedure followed was the same, a slight modification was incorporated by computing the electrostatic and hydrophobic potentials for the atoms of the amino acid residues of the CRD region based on the Conolly molecular surface. Here, the center of the sphere was superimposed with the geometrical center of the surface. The atomic coordinates (x,y,z) of the amino acids were mapped onto a sphere by computing latitude = arcsin(z/|r|) and longitude = arctan(y/x), where r is the radius vector of the atom. The electrostatic potentials and molecular hydrophobicity potential were interpolated on an equal area regular grid on the sphere—latitude [−90°; +90°] and longitude [−180°; +180°] with a 1° step. Mollweide cartographic projection was built to visualize the map using color contours [60]. A modified version of Python script of Koromyslova et al., 2014 [19] was used to map the electrostatic and hydrophobic potentials onto the sphere using scipy.interpolate.griddata, basemap modules and data were visualized using matplotlib.

### (xi) Pearson correlation coefficient to assess conformational changes

Relative similarities and dissimilarities between the different molecular conformations were calculated using Pearson correlation coefficient matrix [61]. The Pearson Coefficient Matrix (r) ranges between -1 and 1, where 1 indicates strong correlation, 0 indicates no relationship between the two variables, value lesser that 0 indicates weak correlation. The correlation coefficient between two mean values f1 and f2 in the two different functions is computed as follows:

$$r=\frac{\sum_{i=1}^{p}(f_{1}(i)-f_{1})(f_{2}-f_{2}(i))}{\sqrt{\sum_{i=1}^{p}{\left(f_{1}-f_{1}(i)\right)}^{2}{\left(f_{2}-f_{2}(i)\right)}^{2}}}$$

Conformational changes in lectins induced due to glycan binding were quantified between the free and bound forms based on solvent accessible surface area (SASA) and residuewise root mean square fluctuations (RMSF) of the CRD and RMSFs in the fourteen key residues. The similarities and dissimilarities are visualized as a square and symmetric matrix with one diagonal and nonzero off-diagonal values representing the Pearson distances in correlation space.

## Supporting information

S1 Table Average RMSD computed for the whole trajectory and sub-trajectories for free and complexed forms of lectins. (DOCX)

S2 Table Average radius of gyration (RG) computed for the whole trajectory and sub-trajectories for free and complexed forms of lectins. (DOCX)

S1 Fig Multiple sequence alignment of the molecular lectin chaperones–calnexin, calreticulin of different species and their homologues in testis specific cells in humans. Sequences enclosed in green box represent lectin domain and red box represent P-domain.
(TIF)

S2 Fig Pictogram represents the docked monoglucosylated-N-glycan (Blue) in the carbohydrate recognition domain (CRD) of lectin (CNXC). (TIF)

S3 Fig Free energy landscapes showing the conformational space of CNXC in free (a–c) and bound forms (e–h): (i) cPCA (a and d); (ii) dPCA-Backbone (b and e); (iii) dPCA-CRD (c and f) and (iv) dPCA-Gly (G). Geometrical separation of the conformational subspace into three distinct clusters using kMeans clustering algorithm (d and i).
(TIF)

S4 Fig Plots showing RMSDs computed as a function of time for Calnexin group of lectins in free form: (A) Calnexin in *Canis lupus* (CNXC); (B) Calnexin in humans (CNXH); and (C) Calmegin (CLMG).
(TIF)

S5 Fig Plots showing RMSDs computed as a function of time for Calreticulin group of lectins in free form: (A) Calreticulin in human (CRTH); (B) Calsperin in humans (CALR3); and (C) Calreticulin in *Entamoeba histolytica* (CRTEh) and (D) Calreticulin in *Trypanozoma cruzi* (CRTTc).
(TIF)

S6 Fig Plots showing RMSDs computed as a function of time for Calnexin group of lectins bound to monoglucosylated-N-glycan: (A) Calnexin in *Canis lupus* (CNXC); (B) Calnexin in humans (CNXH); and (C) Calmegin (CLMG).
(TIF)

S7 Fig Plots showing RMSDs computed as a function of time for Calreticulin group of lectins bound to monoglucosylated-N-glycan: (A) Calreticulin in human (CRTH); (B) Calsperin in humans (CALR3); and (C) Calreticulin in *Entamoeba histolytica* (CRTEh) and (D) Calreticulin in *Trypanozoma cruzi* (CRTTc).
(TIF)

S8 Fig Plots showing radius of gyration (Rg) computed as a function of time for Calnexin group of lectins in free form: (A) Calnexin in *Canis lupus* (CNXC); (B) Calnexin in humans (CNXH); and (C) Calmegin (CLMG).
(TIF)

S9 Fig Plots showing radius of gyration computed as a function of time for Calreticulin group of lectins in free form: (A) Calreticulin in human (CRTH); (B) Calsperin in humans (CALR3); and (C) Calreticulin in *Entamoeba histolytica* (CRTEh) and (D) Calreticulin in *Trypanozoma cruzi* (CRTTc).
(TIF)

S10 Fig Plots showing radius of gyration computed as a function of time for Calnexin group of lectins bound to monoglucosylated-N-glycan: (A) Calnexin in *Canis lupus* (CNXC); (B) Calnexin in humans (CNXH); and (C) Calmegin (CLMG).
(TIF)

S11 Fig Plots showing radius of gyration (Rg) computed as a function of time for Calreticulin group of lectins bound to monoglucosylated-N-glycan: (A) Calreticulin in human (CRTH); (B) Calsperin in humans (CALR3); and (C) Calreticulin in *Entamoeba histolytica* (CRTEh) and (D) Calreticulin in *Trypanozoma cruzi* (CRTTc).
(TIF)

S12 Fig Heatmaps showing the relative similarities among various clusters of free and bound forms of CNXC: (A) SASA; (B) RMSF of CRD and (C) RMSF of conserved residues.
(TIF)

S13 Fig Heatmaps showing the relative similarities among various clusters of free and bound forms of CNXH: (A) SASA; (B) RMSF of CRD and (C) RMSF of conserved residues.
(TIF)

S14 Fig Heatmaps showing the relative similarities among various clusters of free and bound forms of CLMG: (A) SASA; (B) RMSF of CRD and (C) RMSF of conserved residues.
(TIF)

S15 Fig Heatmaps showing the relative similarities among various clusters of free and bound forms of CALR3: (A) SASA; (B) RMSF of CRD and (C) RMSF of conserved residues.
(TIF)

S16 Fig Heatmaps showing the relative similarities among various clusters of free and bound forms of CRTEh: (A) SASA; (B) RMSF of CRD and (C) RMSF of conserved residues.
(TIF)

S17 Fig Heatmaps showing the relative similarities among various clusters of free and bound forms of CRTTc: (A) SASA; (B) RMSF of CRD and (C) RMSF of conserved residues.
(TIF)

S18 Fig Molecular interaction fields of Calnexin in *Canis lupus* (CNXC) with monoglucosylated-N-glycan: (A) Hydrogen bond interactions; (B) Hydrophobic interactions; and (C) van der Waal contacts.
(TIF)

S19 Fig Molecular interaction fields of Calmegin in Humans (CLMG) with monoglucosylated-N-glycan: (A) Hydrogen bond interactions; (B) Hydrophobic interactions; and (C) van der Waal contacts.
(TIF)

S20 Fig Molecular interaction fields of Calreticulin in Humans (CRTH) with monoglucosylated-N-glycan: (A) Hydrogen bond interactions; (B) Hydrophobic interactions; and (C) van der Waal contacts.
(TIF)

S21 Fig Molecular interaction fields of Calsperin in Humans (CALR3) with monoglucosylated-N-glycan: (A) Hydrogen bond interactions; (B) Hydrophobic interactions; and (C) van der Waal contacts.
(TIF)

S22 Fig Molecular interaction fields of Calreticulin in *Entamoeba histolytica* (CRTEh) with monoglucosylated-N-glycan: (A) Hydrogen bond interactions; (B) Hydrophobic interactions; and (C) van der Waal contacts.
(TIF)

S23 Fig Molecular interaction fields of Calreticulin in *Trypanosoma cruzi* (CRTTc) with monoglucosylated-N-glycan: (A) Hydrogen bond interactions; (B) Hydrophobic interactions; and (C) van der Waal contacts.
(TIF)

S24 Fig Protein surface topography showing the MEPs mapped for the free and bound conformers of Calnexin in *Humans* (CNXH): (A) Free-Clus1; (B) Free-Clus2; (C) Free-Clus3; (D) Bound-Clus1; (E) Bound–Clus2; and (C) Bound–Clus3.
(TIF)

S25 Fig Protein surface topography showing the MEPs mapped for the free and bound conformers of Calmegin in Humans (CLMG): (A) Free-Clus1 and (B) Bound-Clus1.
(TIF)

S26 Fig Protein surface topography showing the MEPs mapped for the free and bound conformers of Calsperin in Humans (CALR3): (A) Free-Clus1 and (B) Bound-Clus1.
(TIF)

S27 Fig Protein surface topography showing the MEPs mapped for the free and bound conformers of Calreticulin in *Entamoeba histolytica* (CRTEh) (A) Free-Clus1; (B) Free-Clus2; (C) Free-Clus3; and (D) Bound-Clus1.
(TIF)

S28 Fig Protein surface topography showing the MEPs mapped for the free and bound conformers of Calreticulin in *Trypanosoma cruzi* (CRTTc) (A) Free-Clus1; (B) Free-Clus2; and (C) Bound-Clus1.
(TIF)

S29 Fig Protein surface topography showing the MHPs mapped for the free and bound conformers of Calnexin in *Humans* (CNXH): (A) Free-Clus1; (B) Free-Clus2; (C) Free-Clus3; (D) Bound-Clus1; (E) Bound–Clus2; and (C) Bound–Clus3.
(TIF)

S30 Fig Protein surface topography showing the MHPs mapped for the free and bound conformers of Calmegin in Humans (CLMG): (A) Free-Clus1 and (B) Bound-Clus1.
(TIF)

S31 Fig Protein surface topography showing the MHPs mapped for the free and bound conformers of Calsperin in Humans (CALR3): (A) Free-Clus1 and (B) Bound-Clus1.
(TIF)

S32 Fig Protein surface topography showing the MHPs mapped for the free and bound conformers of Calreticulin in *Entamoeba histolytica* (CRTEh) (A) Free-Clus1; (B) Free-Clus2; (C) Free-Clus3; and (D) Bound-Clus1.
(TIF)

S33 Fig Protein surface topography showing the MHPs mapped for the free and bound conformers of Calreticulin in *Trypanosoma cruzi* (CRTTc) (A) Free-Clus1; (B) Free-Clus2; and (C) Bound-Clus1.
(TIF)

S1 Data Excel workbook listing water mediated hydrogen bonds existing between all the lectin-glycan complexes. (XLSX)

S2 Data Excel workbook listing noncovalent bond interactions observed in different clusters of lectin-glycan complexes. (XLSX)
