# Supplementary material for: Mixed mechanism of conformational selection and induced fit as a molecular recognition process in the calreticulin family of proteins
Source: PLoS Comput Biol. 2022 Dec 12;18(12):e1010661. doi: 10.1371/journal.pcbi.1010661 (PMC9744295; doi:10.1371/journal.pcbi.1010661)
Supplement: S1 Table — (DOCX) [file pcbi.1010661.s001.docx]

**SUPPLEMENTARY TABLES**

**S1 Table: Average RMSD computed for the whole trajectory and sub-trajectories for free and complexed forms of lectins**

|  | Protein | Whole Trajectory | CLUS1 | CLUS2 | CLUS3 | CLUS4 |
| --- | --- | --- | --- | --- | --- | --- |
|  | CNXC | 0.35 ± 0.04 | 0.41 ± 0.03 | 0.35 ± 0.02 | 0.35 ± 0.03 | - |
|  | CNXH | 0.30 ± 0.03 | 0.31 ± 0.01 | 0.29 ± 0.01 | 0.32 ± 0.01 | - |
| Free | CMG | 0.28 ± 0.02 | 0.28 ± 0.02 | - | - | - |
|  | CRTH | 0.30 ± 0.03 | 0.31 ± 0.01 | 0.29 ± 0.01 | 0.32 ± 0.01 | 0.32 ± 0.08 |
|  | CLSP | 0.28 ± 0.29 | 0.29 ± 0.02 | - | - | - |
|  | CRTEh | 0.17 ± 0.03 | 0.14 ± 0.02 | 0.18 ± 0.02 | 0.20 ± 0.02 | - |
|  | CRTTc | 0.17 ± 0.03 | 0.21 ± 0.01 | 0.13 ± 0.01 | - | - |
|  | CNXC | 0.34 ± 0.07 | 0.38 ± 0.07 | 0.43 ± 0.03 | 0.31 ± 0.02 | - |
|  | CNXH | 0.47 ± 0.04 | 0.48 ± 0.03 | 0.43 ± 0.02 | 0.50 ± 0.02 | - |
|  | CMG | 0.47 ± 0.08 | 0.48 ± 0.02 | - | - | - |
| Complex | CRTH | 0.36 ± 0.07 | 0.32 ± 0.04 | 0.34 ± 0.06 | 0.40 ± 0.05 | 0.38 ± 0.05 |
|  | CLSP | 0.58 ± 0.09 | 0.54 ± 0.06 | - | - | - |
|  | CRTEh | 0.61 ± 0.04 | 0.61 ± 0.02 | - | - | - |
|  | CRTTc | 0.20 ± 0.05 | 0.19 ± 0.02 | - | - | - |
