# Supplementary material for: Mixed mechanism of conformational selection and induced fit as a molecular recognition process in the calreticulin family of proteins
Source: PLoS Comput Biol. 2022 Dec 12;18(12):e1010661. doi: 10.1371/journal.pcbi.1010661 (PMC9744295; doi:10.1371/journal.pcbi.1010661)
Supplement: S2 Table — (DOCX) [file pcbi.1010661.s002.docx]

**S2 Table: Average radius of gyration (RG) computed for the whole trajectory and sub-trajectories for free and complexed forms of lectins**

|  | Protein | Whole Trajectory | CLUS1 | CLUS2 | CLUS3 | CLUS4 |
| --- | --- | --- | --- | --- | --- | --- |
|  | CNXC | 1.79 ± 0.01 | 1.79 ± 0.01 | 1.78 ± 0.01 | 1.80 ± 0.00 | - |
|  | CNXH | 1.76 ± 0.01 | 1.75 ± 0.00 | 1.75 ± 0.00 | 1.75 ± 0.00 | - |
| Free | CMG | 1.79 ± 0.01 | 1.79 ± 0.00 | - | - | - |
|  | CRTH | 1.81 ± 0.02 | 1.81 ± 0.00 | 1.78 ± 0.01 | 1.83 ± 0.01 | 1.83 ± 0.01 |
|  | CLSP | 1.78 ± 0.01 | 1.78 ± 0.00 | - | - | - |
|  | CRTEh | 1.72 ± 0.01 | 1.72 ± 0.00 | 1.72 ± 0.00 | 1.71 ± 0.01 | - |
|  | CRTTc | 1.71 ± 0.01 | 1.72 ± 0.00 | 1.70 ± 0.00 | - | - |
|  | CNXC | 1.89 ± 0.01 | 1.89 ± 0.01 | 1.88 ± 0.01 | 1.89 ± 0.00 | - |
|  | CNXH | 1.85 ± 0.01 | 1.84 ± 0.01 | 1.87 ± 0.01 | 1.85 ± 0.00 | - |
|  | CMG | 1.88 ± 0.02 | 1.88 ± 0.01 | - | - | - |
| Complex | CRTH | 1.88 ± 0.02 | 1.88 ± 0.02 | 1.87 ± 0.02 | 1.88 ± 0.02 | 1.87 ± 0.01 |
|  | CLSP | 1.90 ± 0.02 | 1.93 ± 0.02 | - | - | - |
|  | CRTEh | 1.87 ± 0.01 | 1.87 ± 0.01 | - | - | - |
|  | CRTTc | 1.80 ± 0.01 | 1.79 ± 0.00 | - | - | - |
