# Supplementary figures and images for: Mixed mechanism of conformational selection and induced fit as a molecular recognition process in the calreticulin family of proteins
Source: PLoS Comput Biol. 2022 Dec 12;18(12):e1010661. doi: 10.1371/journal.pcbi.1010661 (PMC9744295; doi:10.1371/journal.pcbi.1010661)

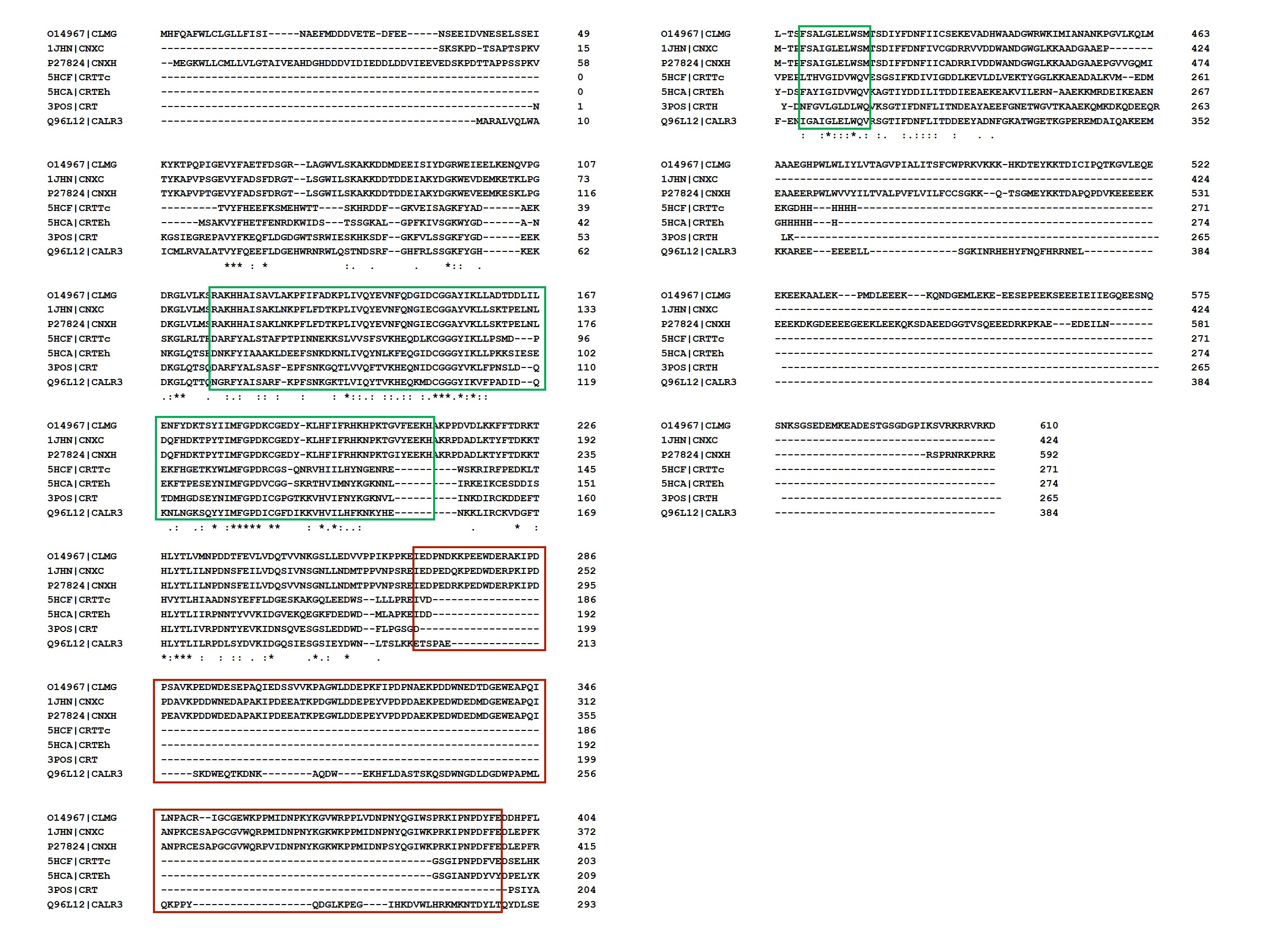

Supplement: S1 Fig — Sequences enclosed in green box represent lectin domain and red box represent P-domain. (TIF) [file pcbi.1010661.s003.tif]

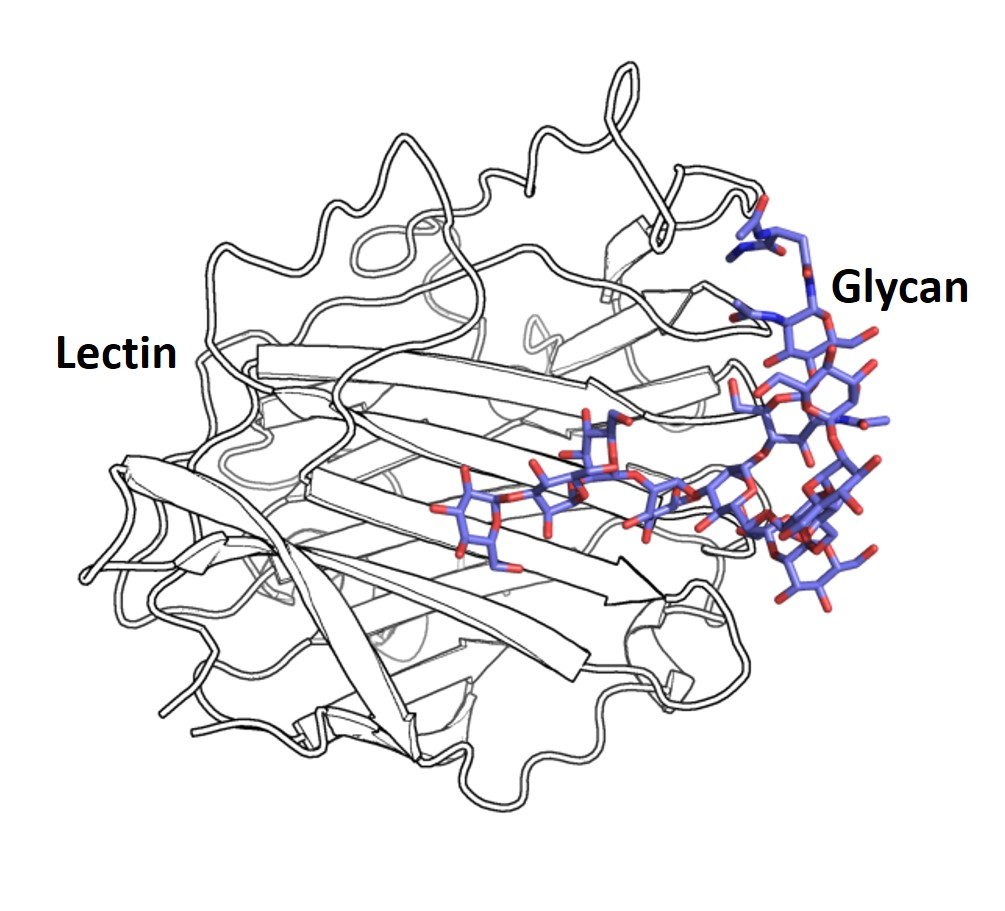

Supplement: S2 Fig — (TIF) [file pcbi.1010661.s004.tif]

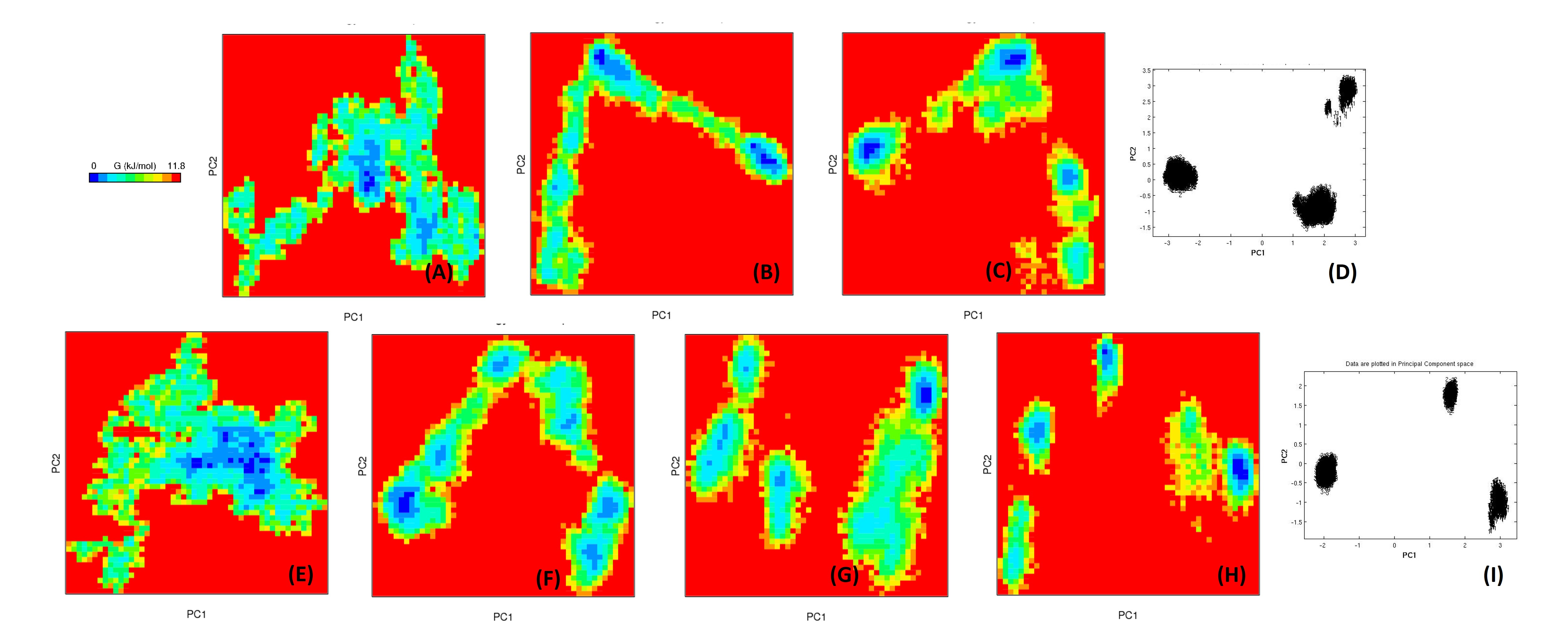

Supplement: S3 Fig — Free energy landscapes showing the conformational space of CNXC in free (a–c) and bound forms (e–h): (i) cPCA (a and d); (ii) dPCA-Backbone (b and e); (iii) dPCA-CRD (c and f) and (iv) dPCA-Gly (G). Geometrical separation of the conformational subspace into three distinct clusters using kMeans clustering algorithm (d and i). (TIF) [file pcbi.1010661.s005.tif]

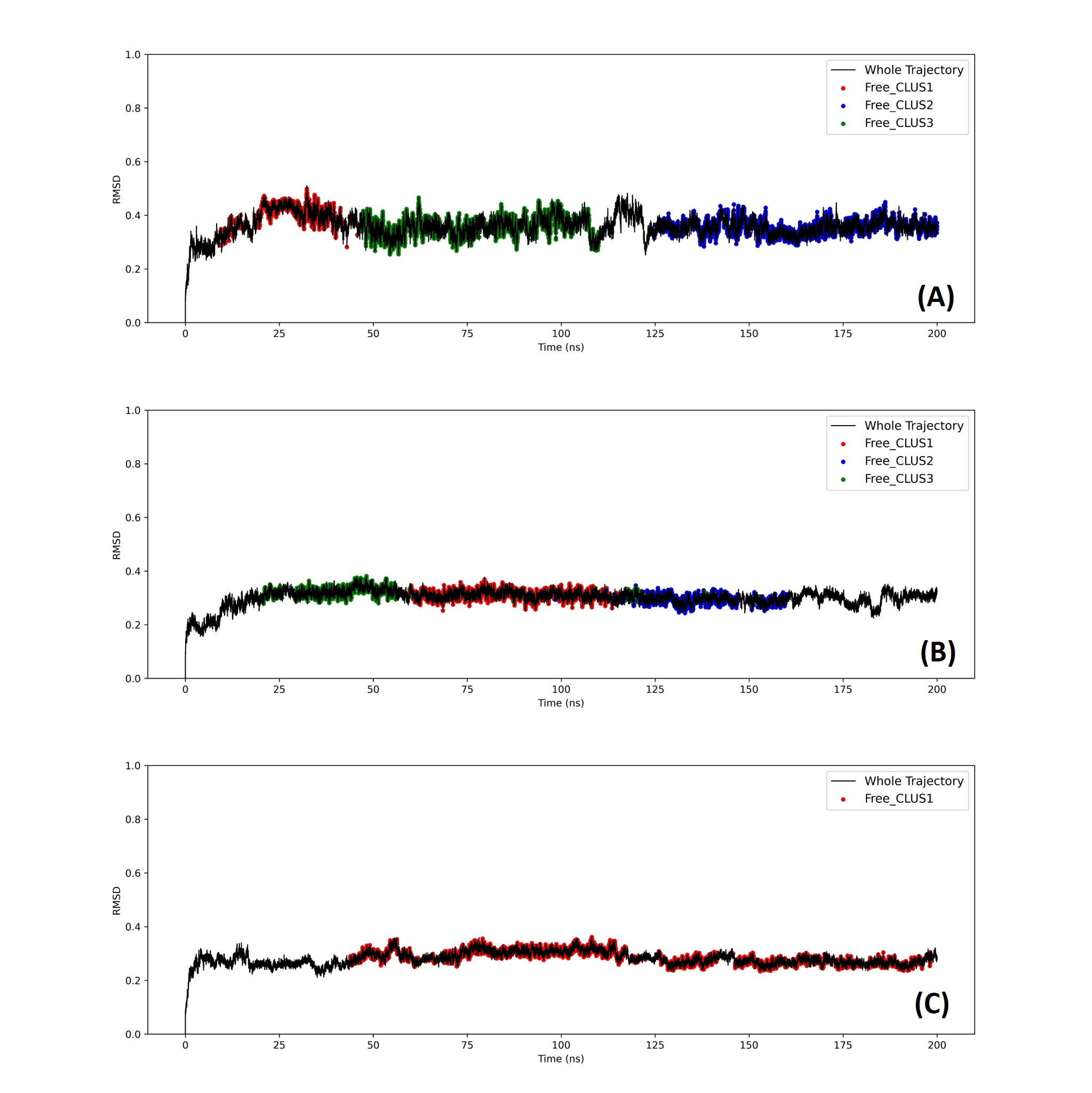

Supplement: S4 Fig — Plots showing RMSDs computed as a function of time for Calnexin group of lectins in free form: (A) Calnexin in Canis lupus (CNXC); (B) Calnexin in humans (CNXH); and (C) Calmegin (CLMG). (TIF) [file pcbi.1010661.s006.tif]

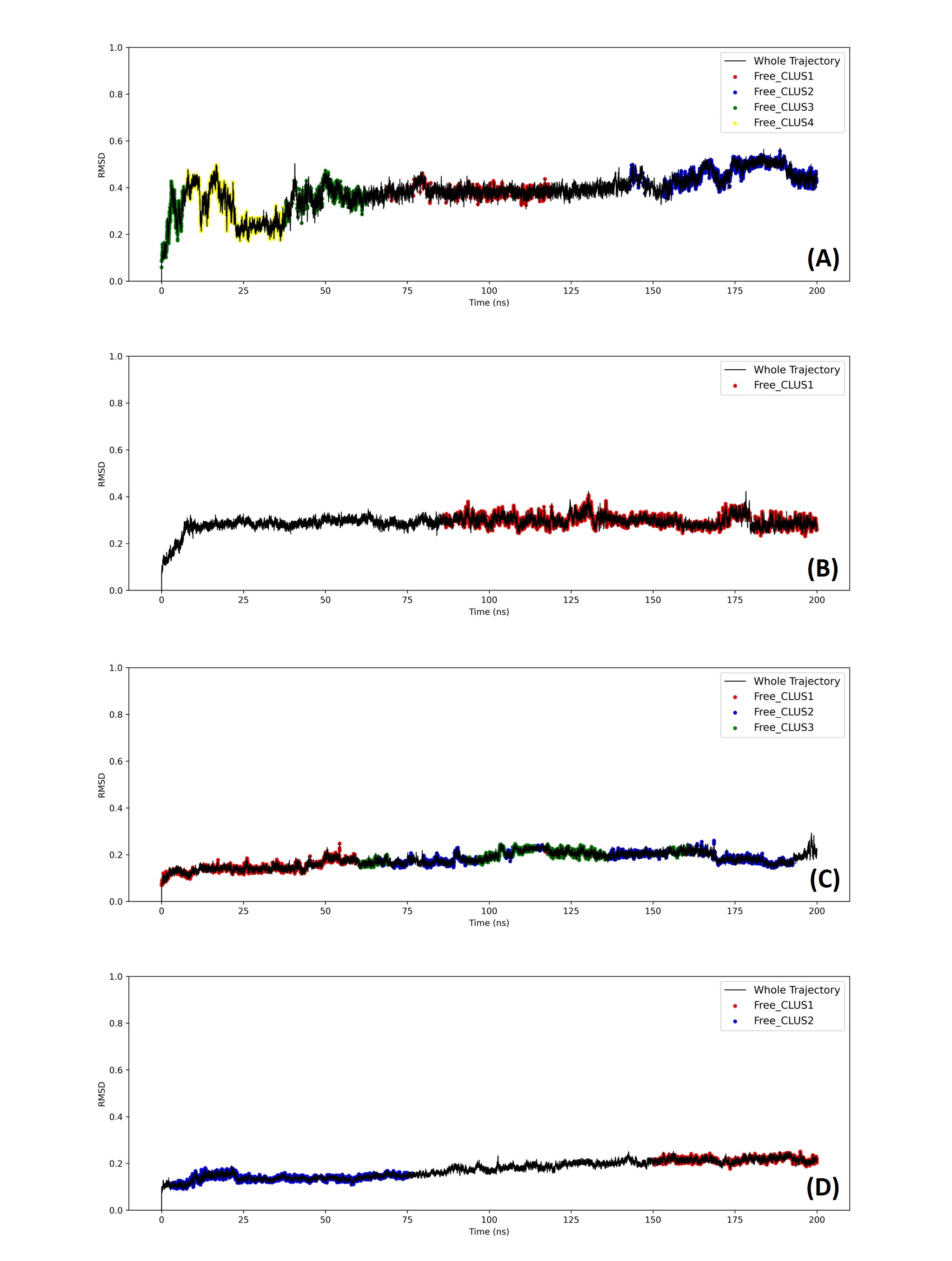

Supplement: S5 Fig — Plots showing RMSDs computed as a function of time for Calreticulin group of lectins in free form: (A) Calreticulin in human (CRTH); (B) Calsperin in humans (CALR3); and (C) Calreticulin in Entamoeba histolytica (CRTEh) and (D) Calreticulin in Trypanozoma cruzi (CRTTc). (TIF) [file pcbi.1010661.s007.tif]

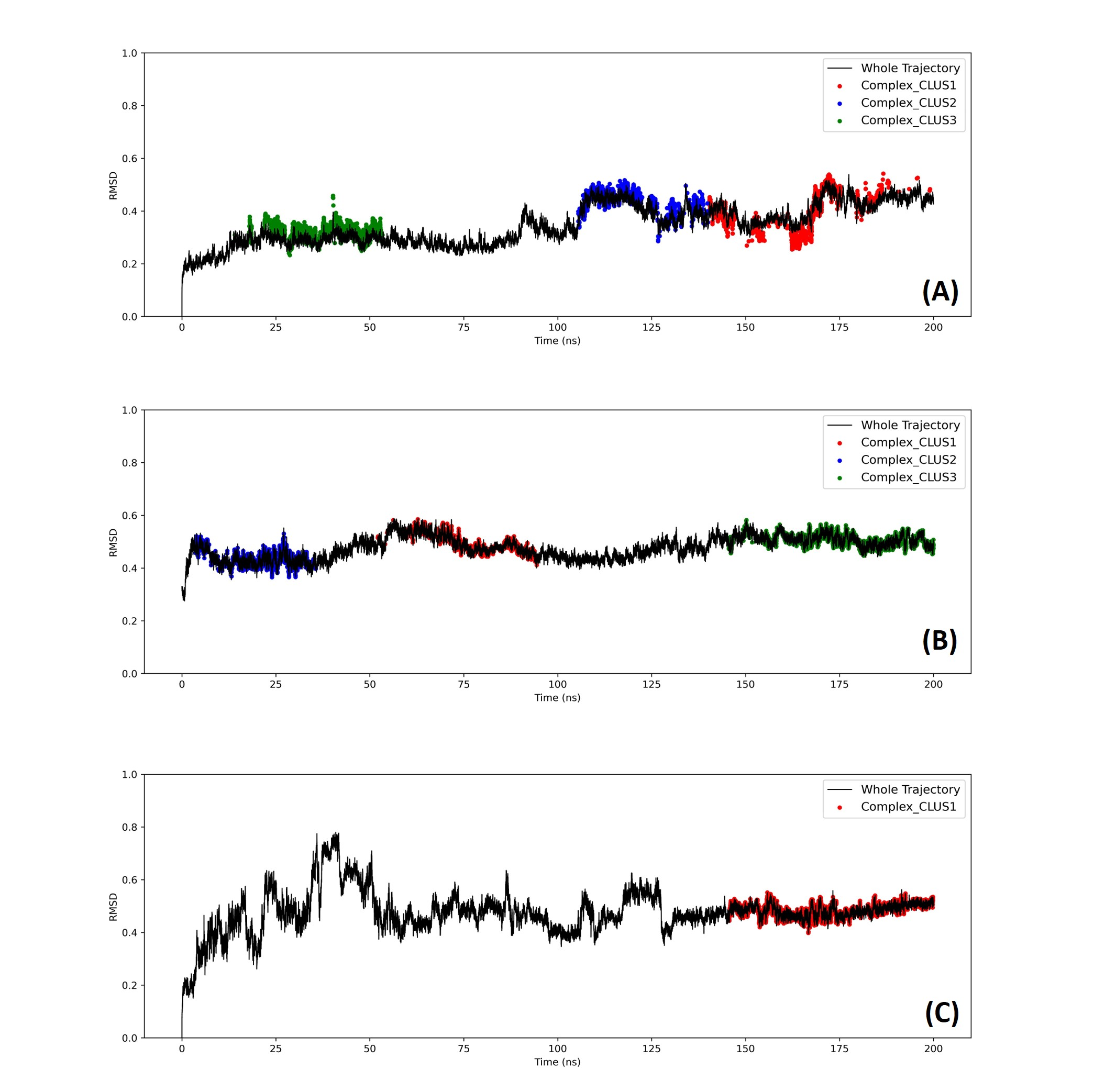

Supplement: S6 Fig — Plots showing RMSDs computed as a function of time for Calnexin group of lectins bound to monoglucosylated-N-glycan: (A) Calnexin in Canis lupus (CNXC); (B) Calnexin in humans (CNXH); and (C) Calmegin (CLMG). (TIF) [file pcbi.1010661.s008.tif]

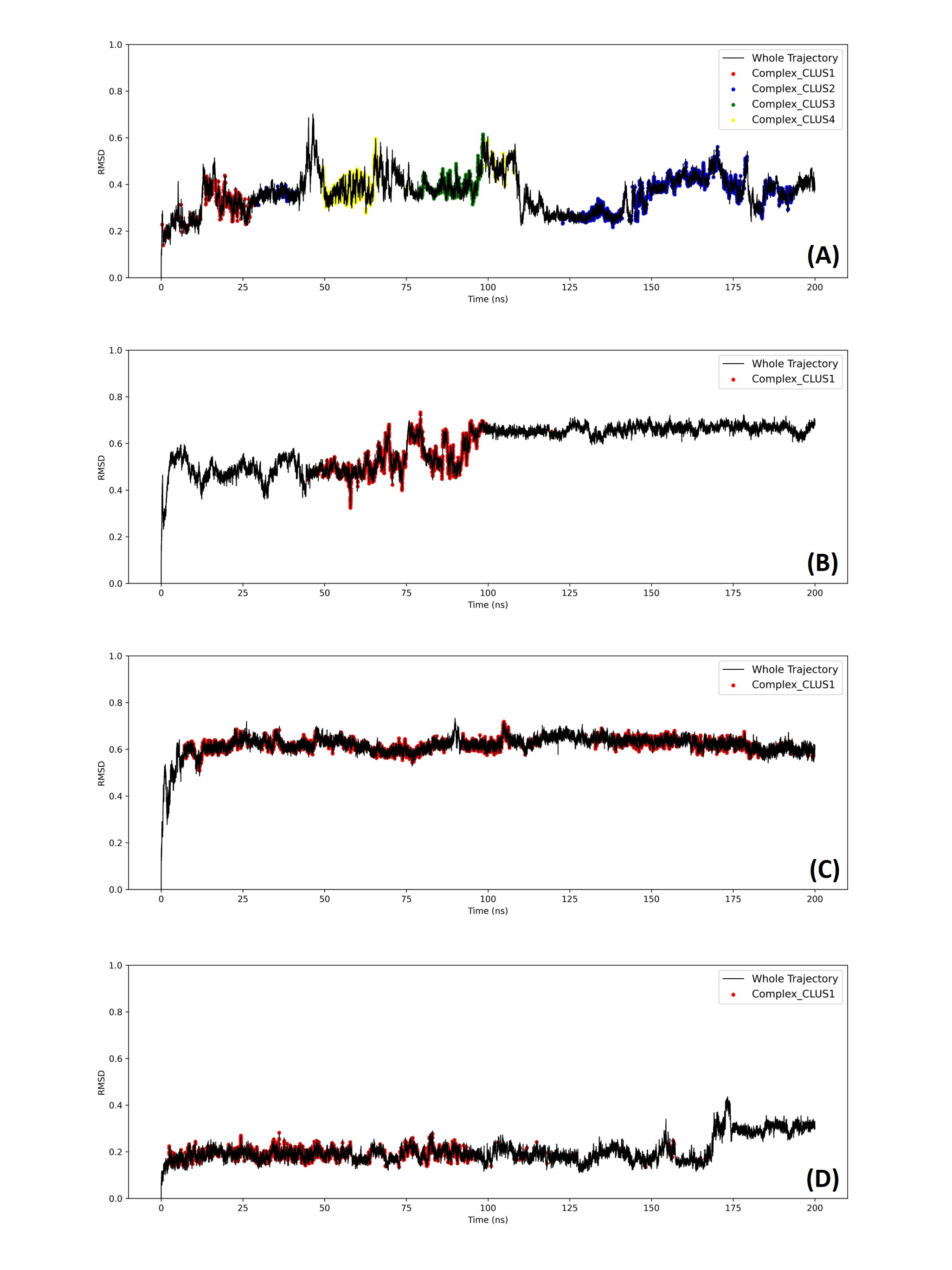

Supplement: S7 Fig — Plots showing RMSDs computed as a function of time for Calreticulin group of lectins bound to monoglucosylated-N-glycan: (A) Calreticulin in human (CRTH); (B) Calsperin in humans (CALR3); and (C) Calreticulin in Entamoeba histolytica (CRTEh) and (D) Calreticulin in Trypanozoma cruzi (CRTTc). (TIF) [file pcbi.1010661.s009.tif]

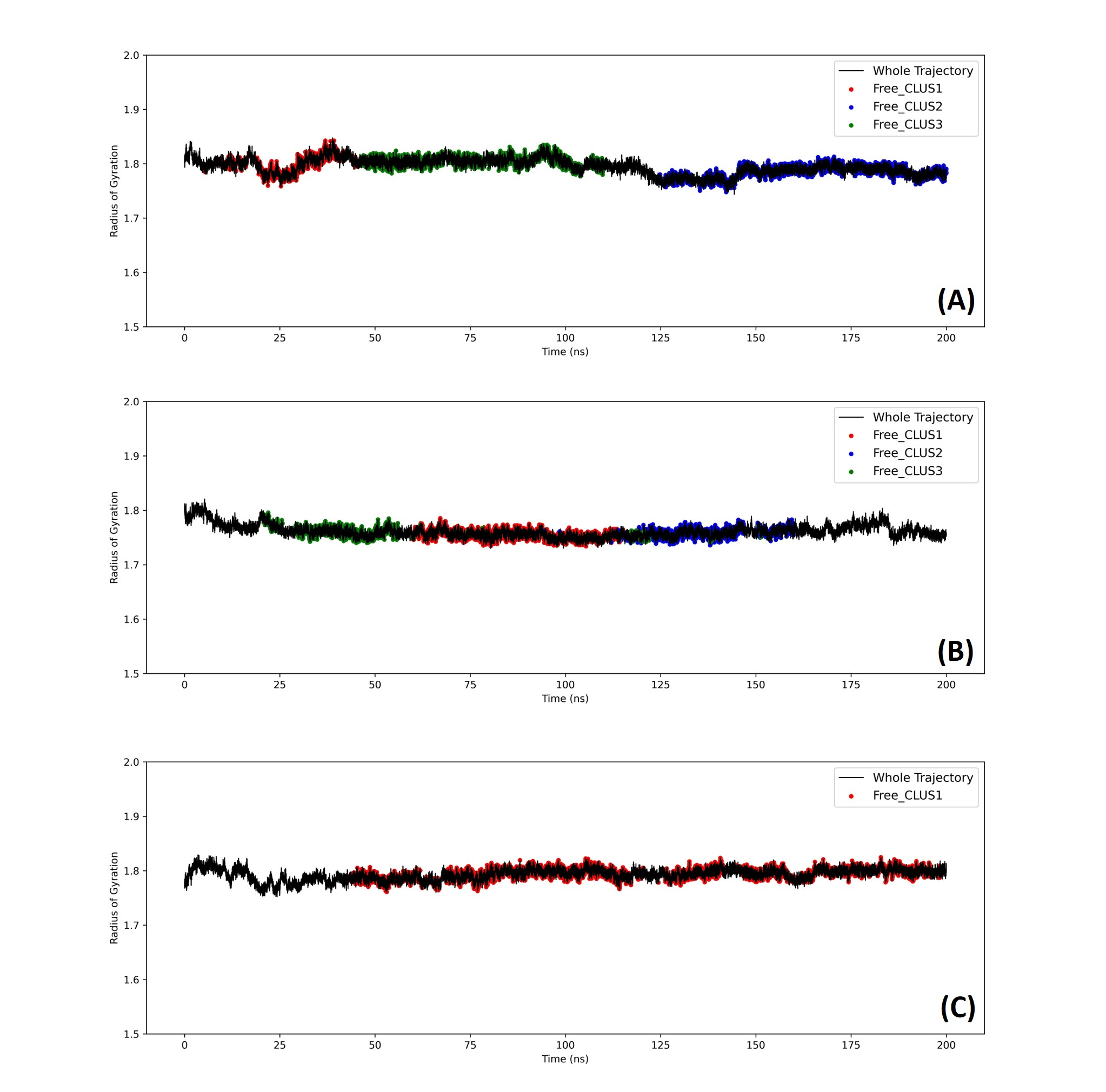

Supplement: S8 Fig — Plots showing radius of gyration (Rg) computed as a function of time for Calnexin group of lectins in free form: (A) Calnexin in Canis lupus (CNXC); (B) Calnexin in humans (CNXH); and (C) Calmegin (CLMG). (TIF) [file pcbi.1010661.s010.tif]

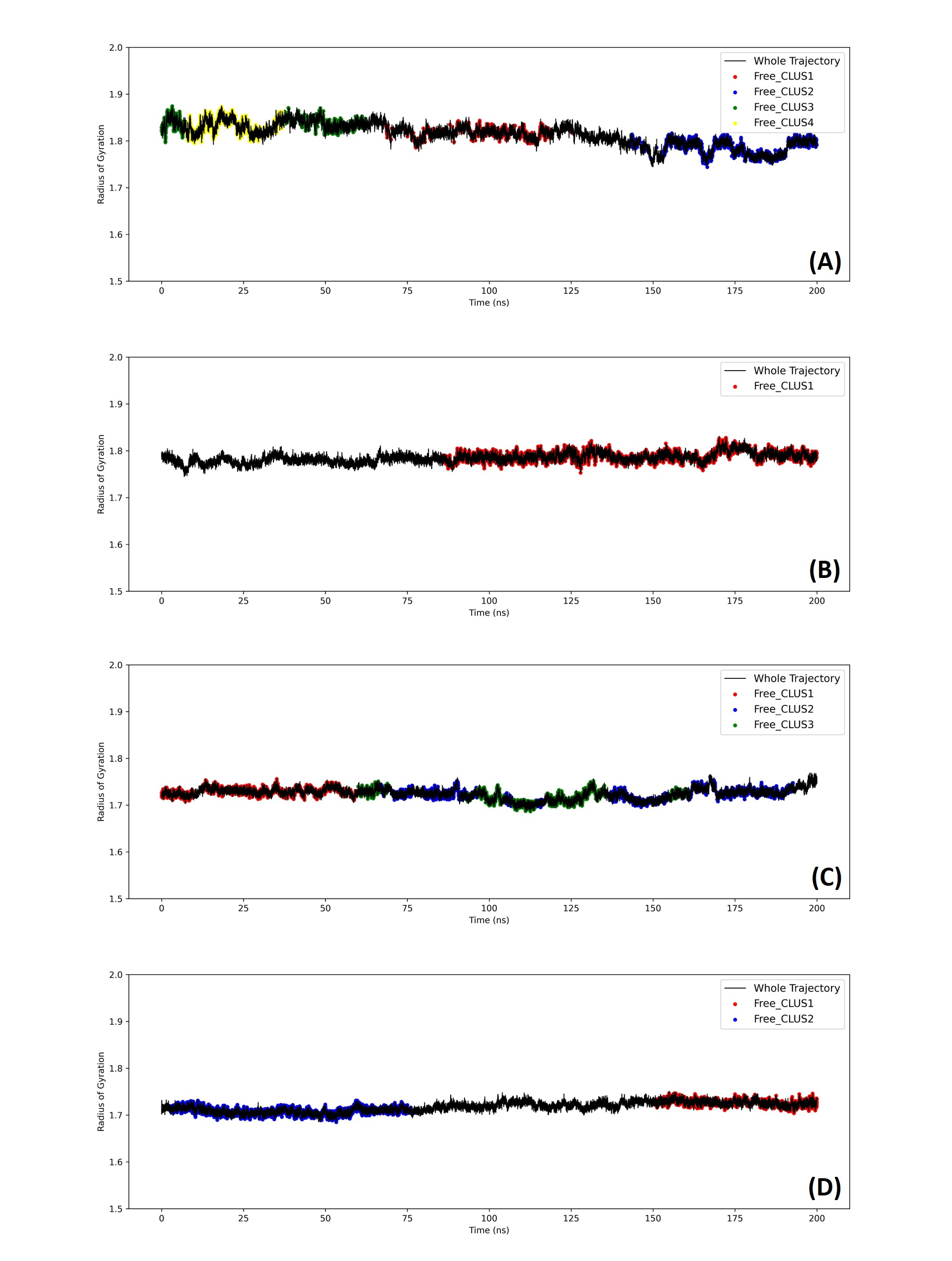

Supplement: S9 Fig — Plots showing radius of gyration computed as a function of time for Calreticulin group of lectins in free form: (A) Calreticulin in human (CRTH); (B) Calsperin in humans (CALR3); and (C) Calreticulin in Entamoeba histolytica (CRTEh) and (D) Calreticulin in Trypanozoma cruzi (CRTTc). (TIF) [file pcbi.1010661.s011.tif]

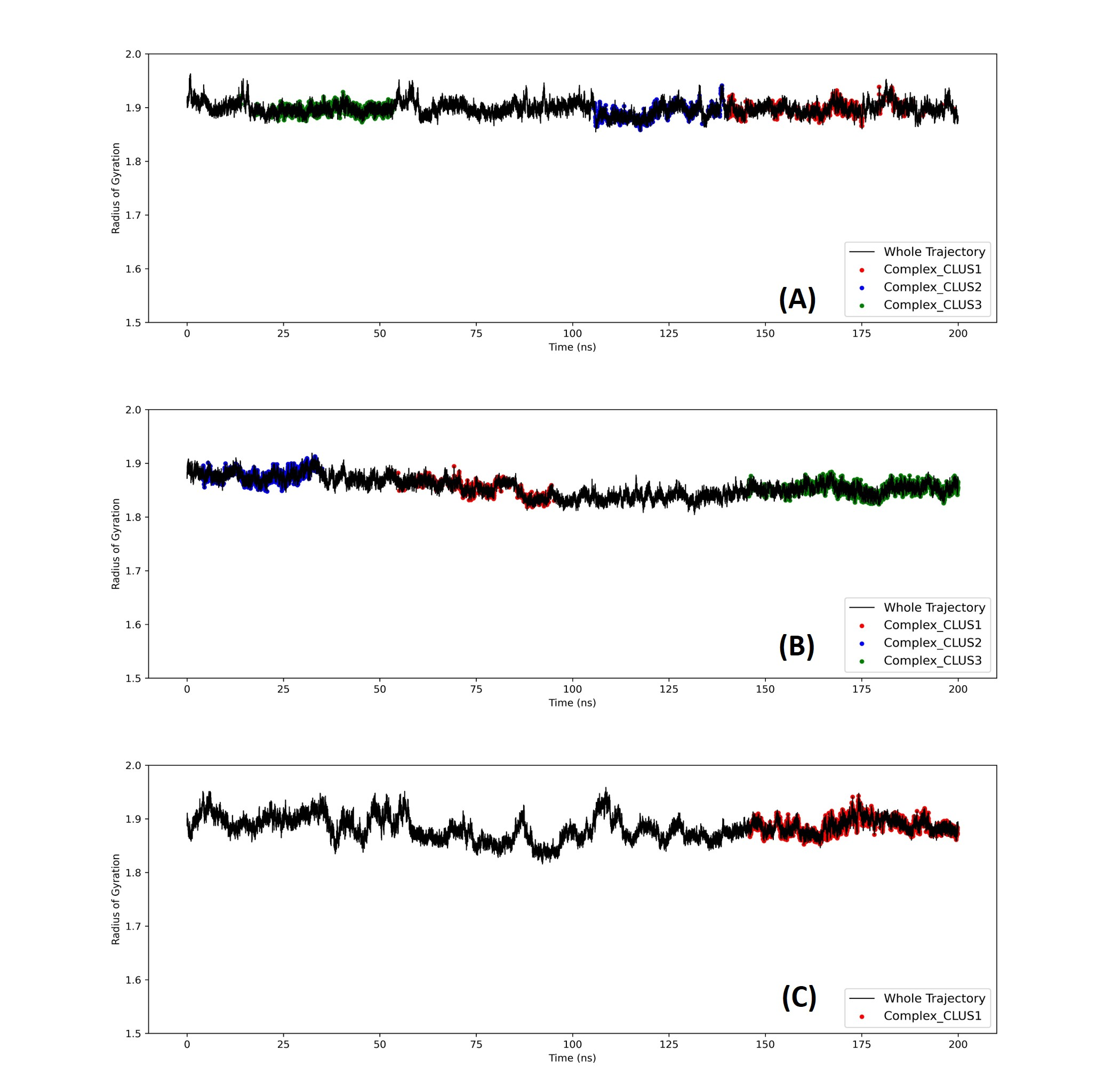

Supplement: S10 Fig — Plots showing radius of gyration computed as a function of time for Calnexin group of lectins bound to monoglucosylated-N-glycan: (A) Calnexin in Canis lupus (CNXC); (B) Calnexin in humans (CNXH); and (C) Calmegin (CLMG). (TIF) [file pcbi.1010661.s012.tif]

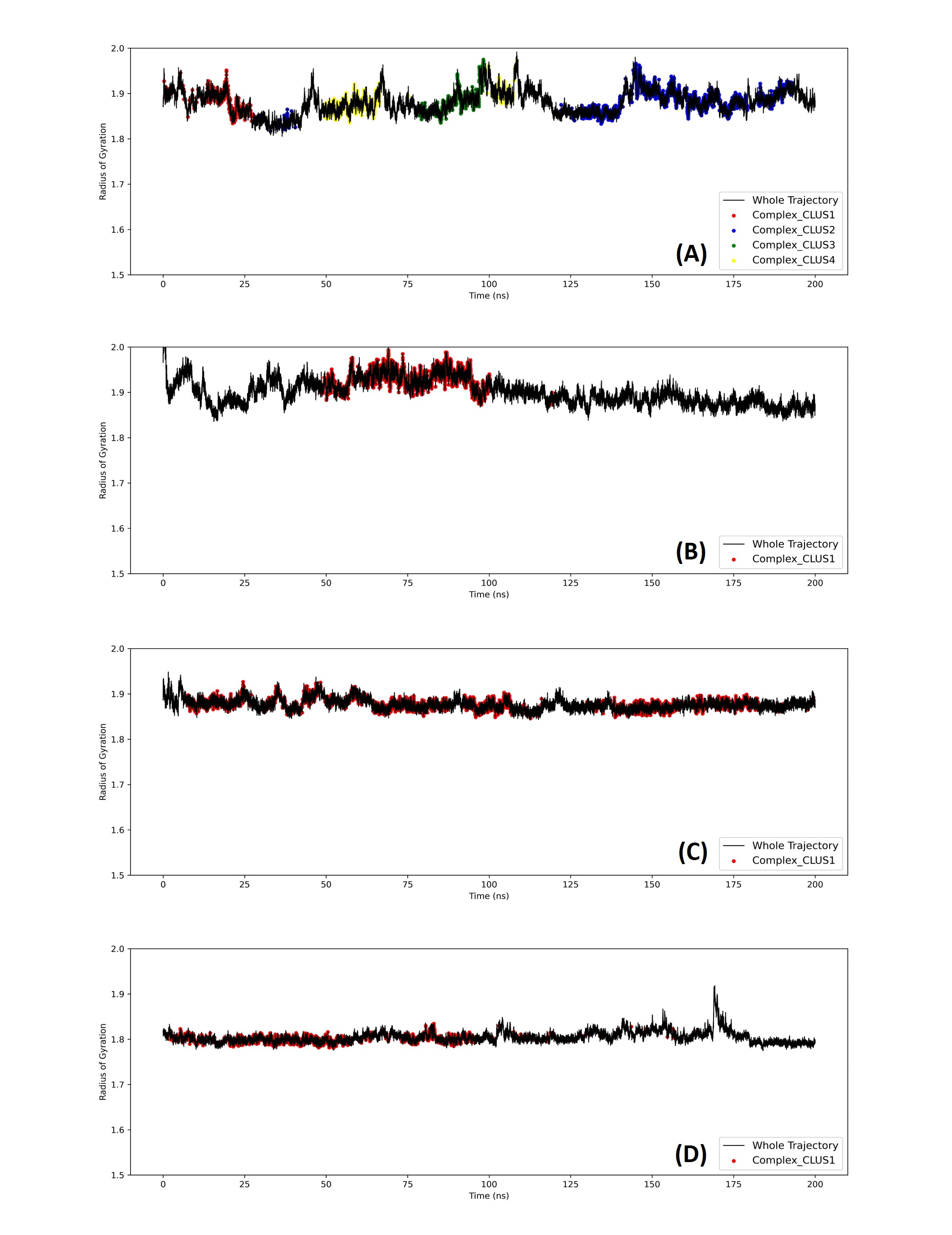

Supplement: S11 Fig — Plots showing radius of gyration (Rg) computed as a function of time for Calreticulin group of lectins bound to monoglucosylated-N-glycan: (A) Calreticulin in human (CRTH); (B) Calsperin in humans (CALR3); and (C) Calreticulin in Entamoeba histolytica (CRTEh) and (D) Calreticulin in Trypanozoma cruzi (CRTTc). (TIF) [file pcbi.1010661.s013.tif]

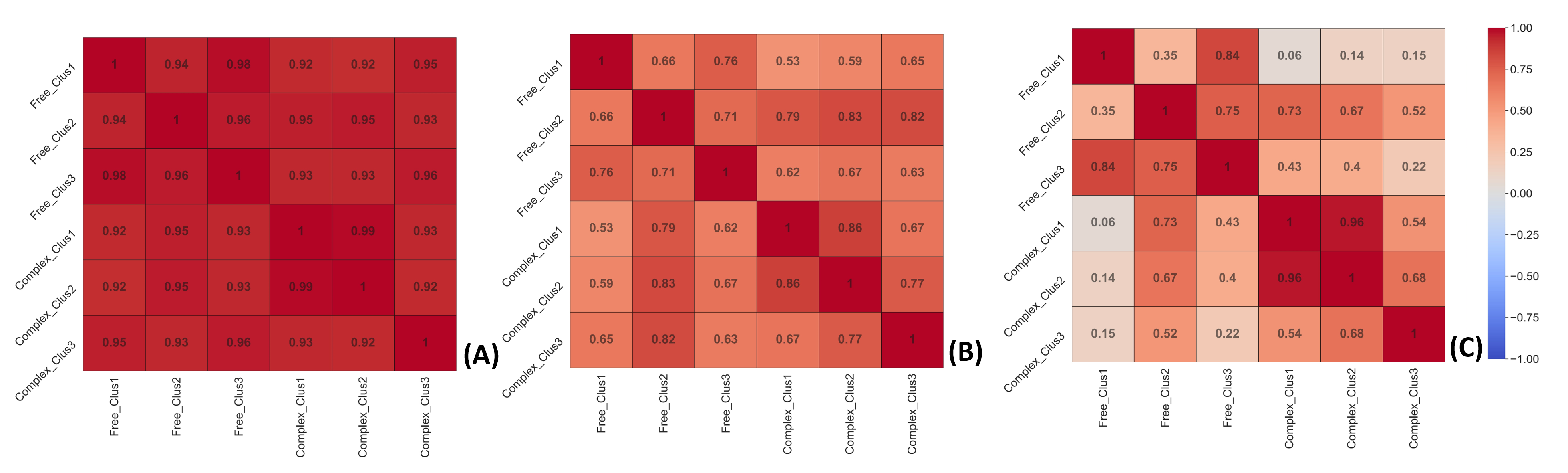

Supplement: S12 Fig — Heatmaps showing the relative similarities among various clusters of free and bound forms of CNXC: (A) SASA; (B) RMSF of CRD and (C) RMSF of conserved residues. (TIF) [file pcbi.1010661.s014.tif]

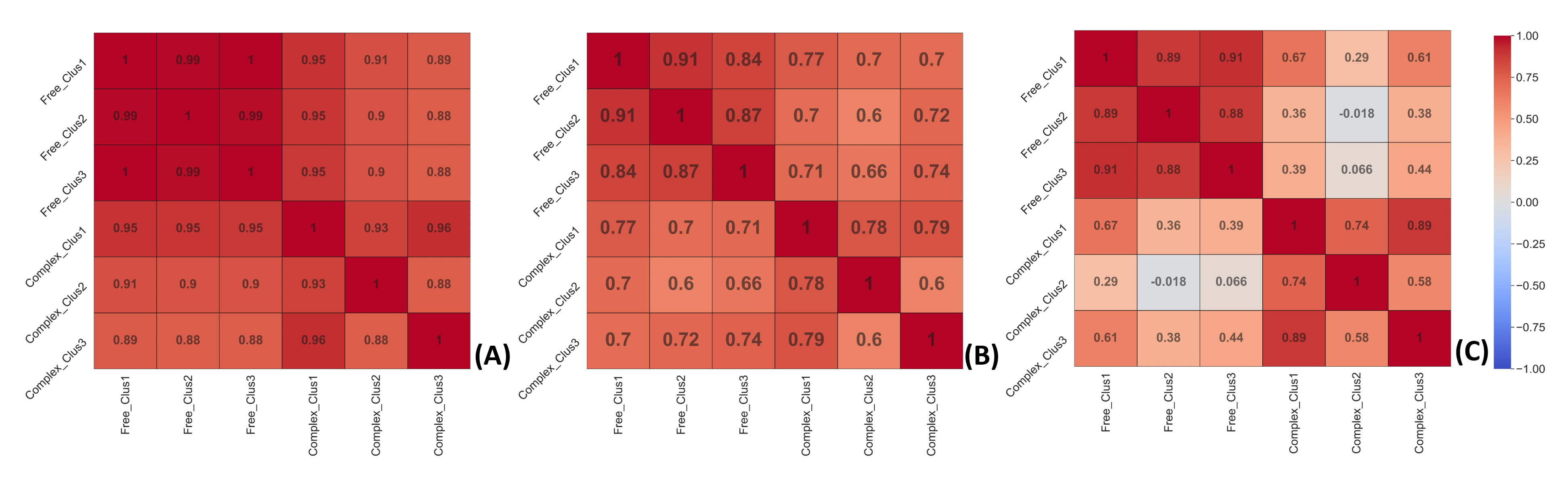

Supplement: S13 Fig — Heatmaps showing the relative similarities among various clusters of free and bound forms of CNXH: (A) SASA; (B) RMSF of CRD and (C) RMSF of conserved residues. (TIF) [file pcbi.1010661.s015.tif]

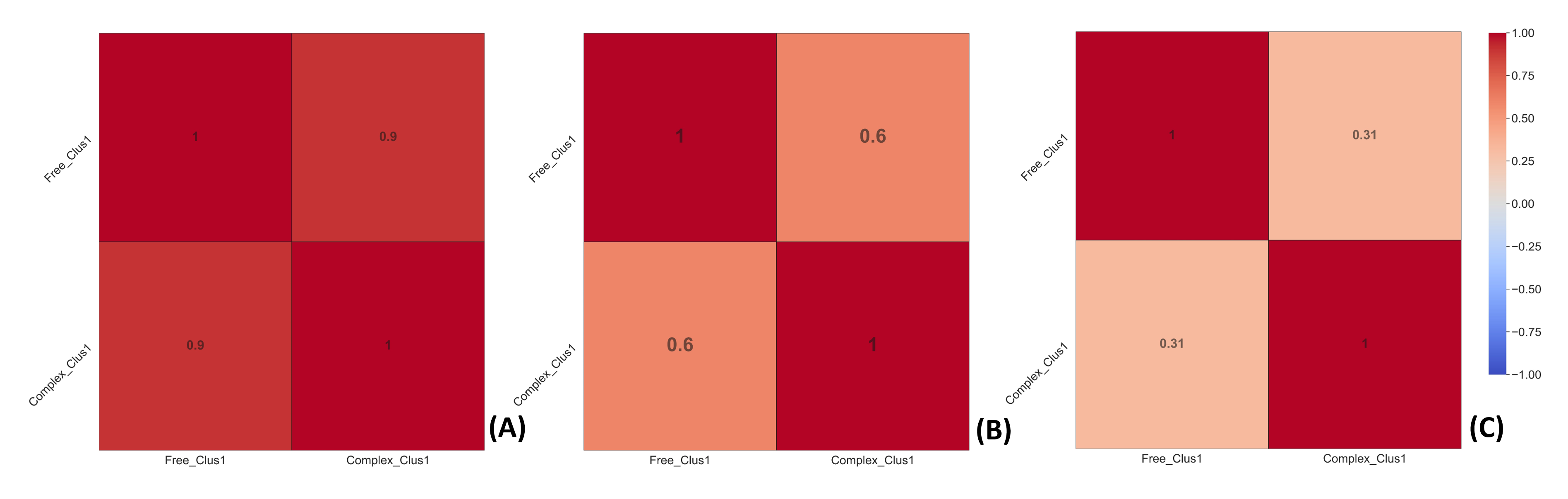

Supplement: S14 Fig — Heatmaps showing the relative similarities among various clusters of free and bound forms of CLMG: (A) SASA; (B) RMSF of CRD and (C) RMSF of conserved residues. (TIF) [file pcbi.1010661.s016.tif]

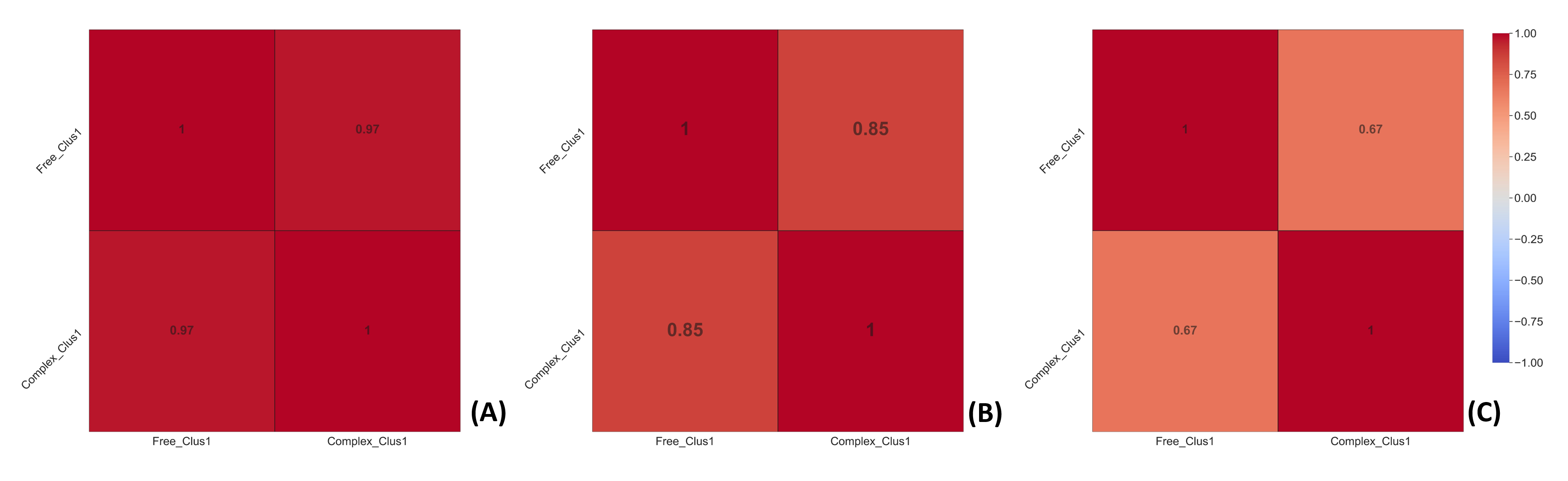

Supplement: S15 Fig — Heatmaps showing the relative similarities among various clusters of free and bound forms of CALR3: (A) SASA; (B) RMSF of CRD and (C) RMSF of conserved residues. (TIF) [file pcbi.1010661.s017.tif]

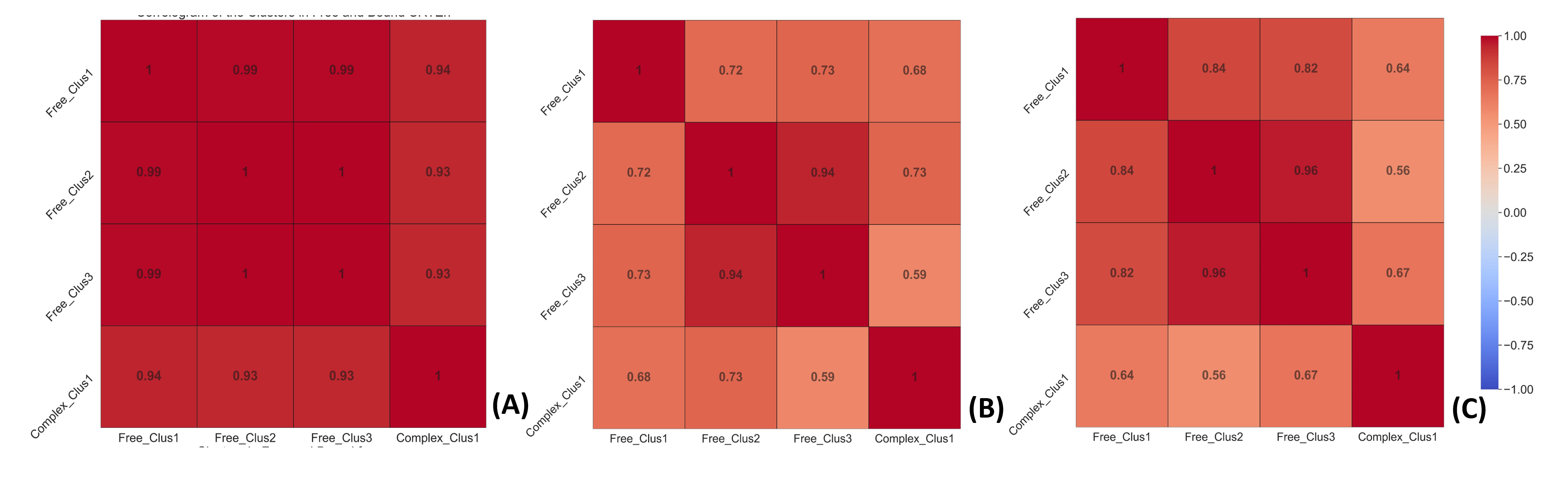

Supplement: S16 Fig — Heatmaps showing the relative similarities among various clusters of free and bound forms of CRTEh: (A) SASA; (B) RMSF of CRD and (C) RMSF of conserved residues. (TIF) [file pcbi.1010661.s018.tif]

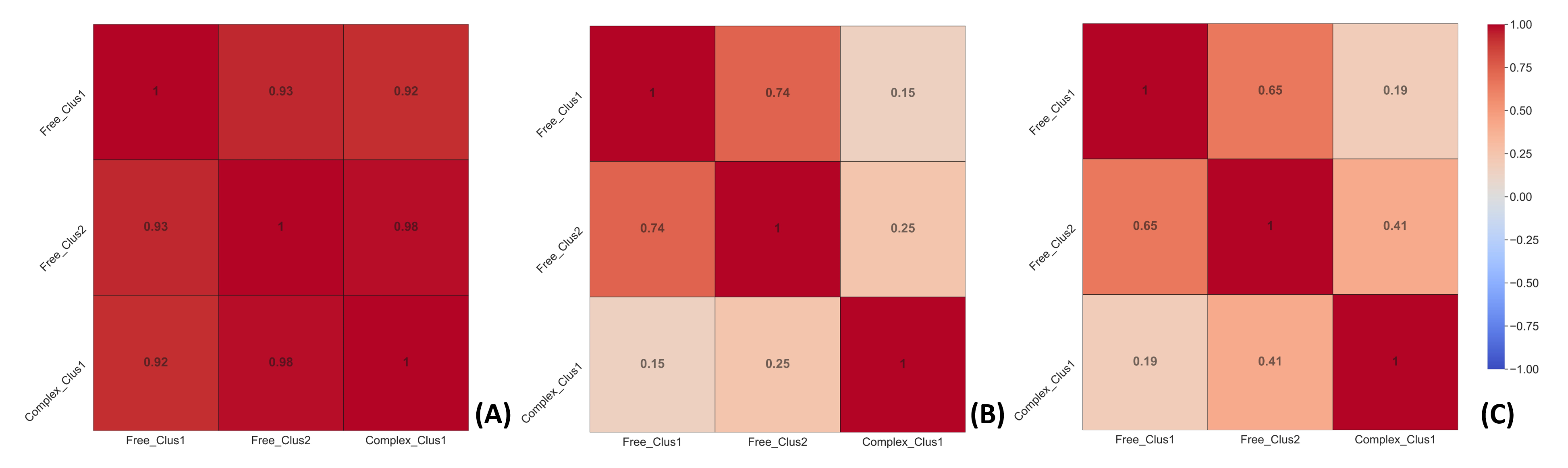

Supplement: S17 Fig — Heatmaps showing the relative similarities among various clusters of free and bound forms of CRTTc: (A) SASA; (B) RMSF of CRD and (C) RMSF of conserved residues. (TIF) [file pcbi.1010661.s019.tif]

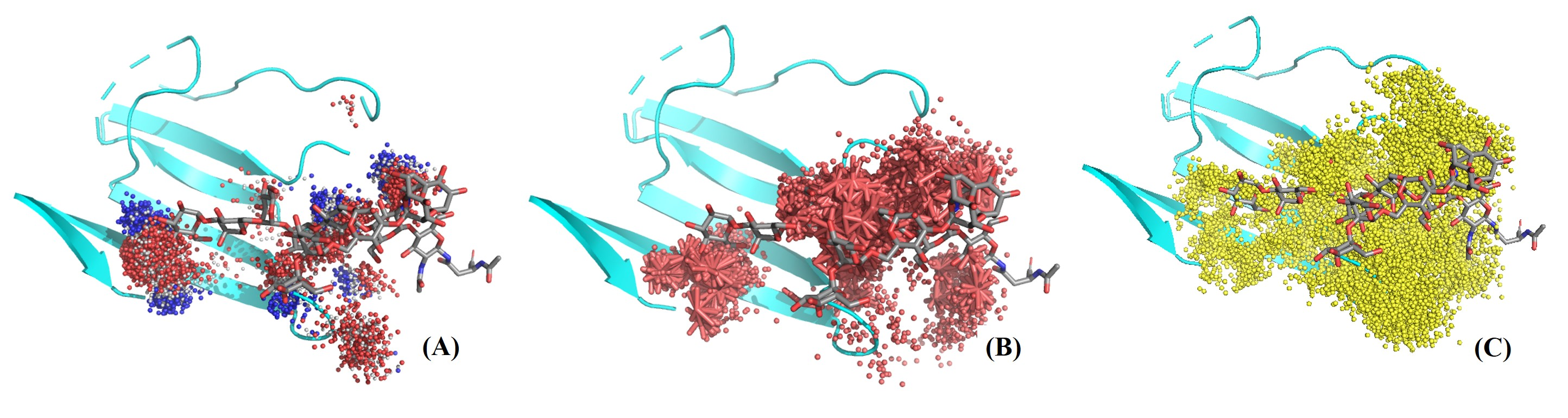

Supplement: S18 Fig — Molecular interaction fields of Calnexin in Canis lupus (CNXC) with monoglucosylated-N-glycan: (A) Hydrogen bond interactions; (B) Hydrophobic interactions; and (C) van der Waal contacts. (TIF) [file pcbi.1010661.s020.tif]

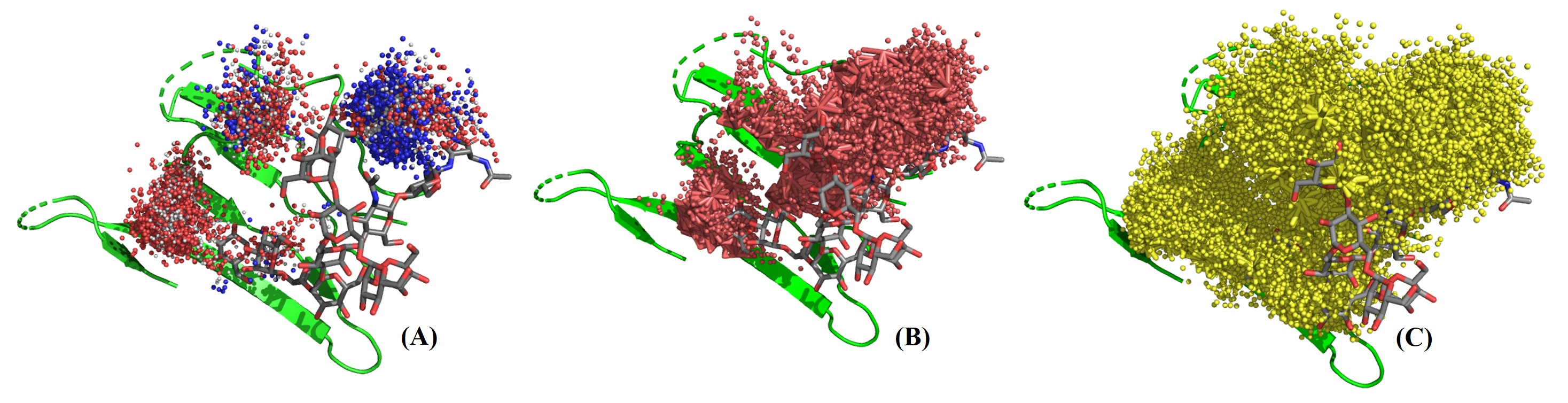

Supplement: S19 Fig — Molecular interaction fields of Calmegin in Humans (CLMG) with monoglucosylated-N-glycan: (A) Hydrogen bond interactions; (B) Hydrophobic interactions; and (C) van der Waal contacts. (TIF) [file pcbi.1010661.s021.tif]

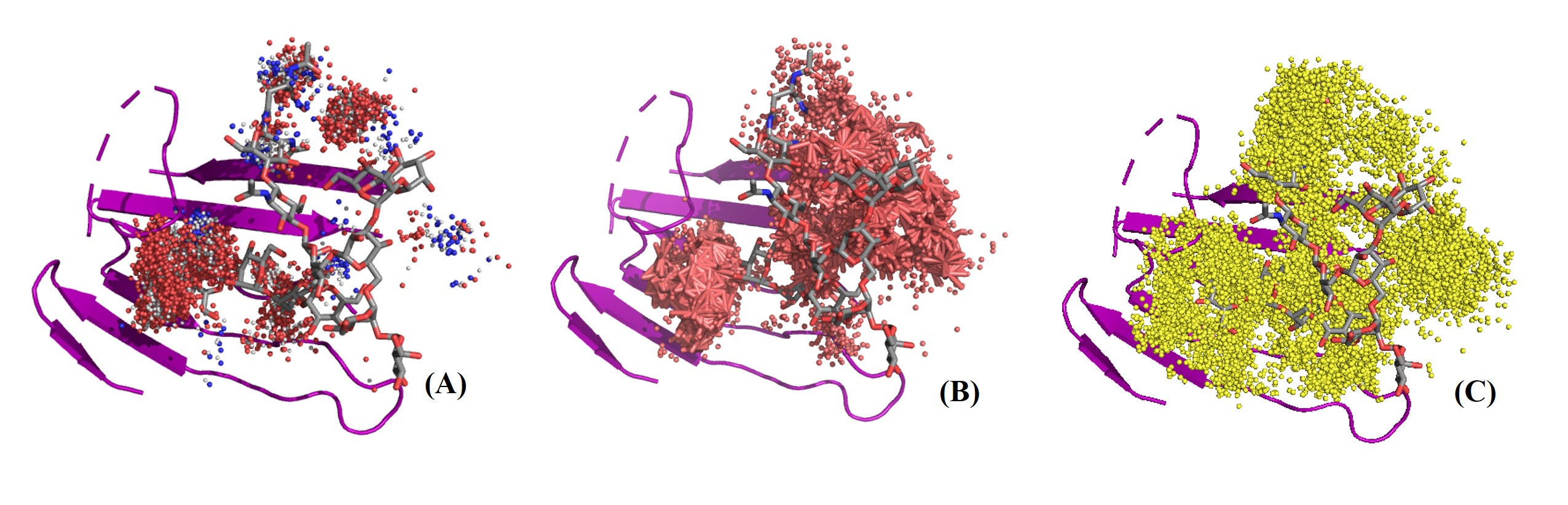

Supplement: S20 Fig — Molecular interaction fields of Calreticulin in Humans (CRTH) with monoglucosylated-N-glycan: (A) Hydrogen bond interactions; (B) Hydrophobic interactions; and (C) van der Waal contacts. (TIF) [file pcbi.1010661.s022.tif]

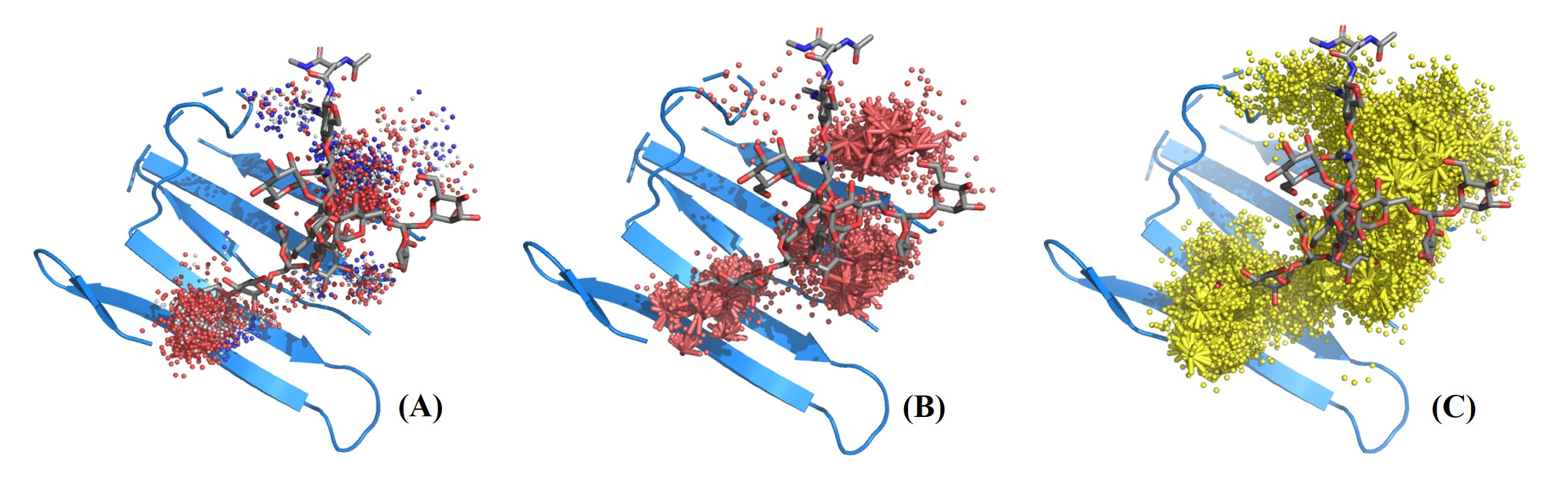

Supplement: S21 Fig — Molecular interaction fields of Calsperin in Humans (CALR3) with monoglucosylated-N-glycan: (A) Hydrogen bond interactions; (B) Hydrophobic interactions; and (C) van der Waal contacts. (TIF) [file pcbi.1010661.s023.tif]

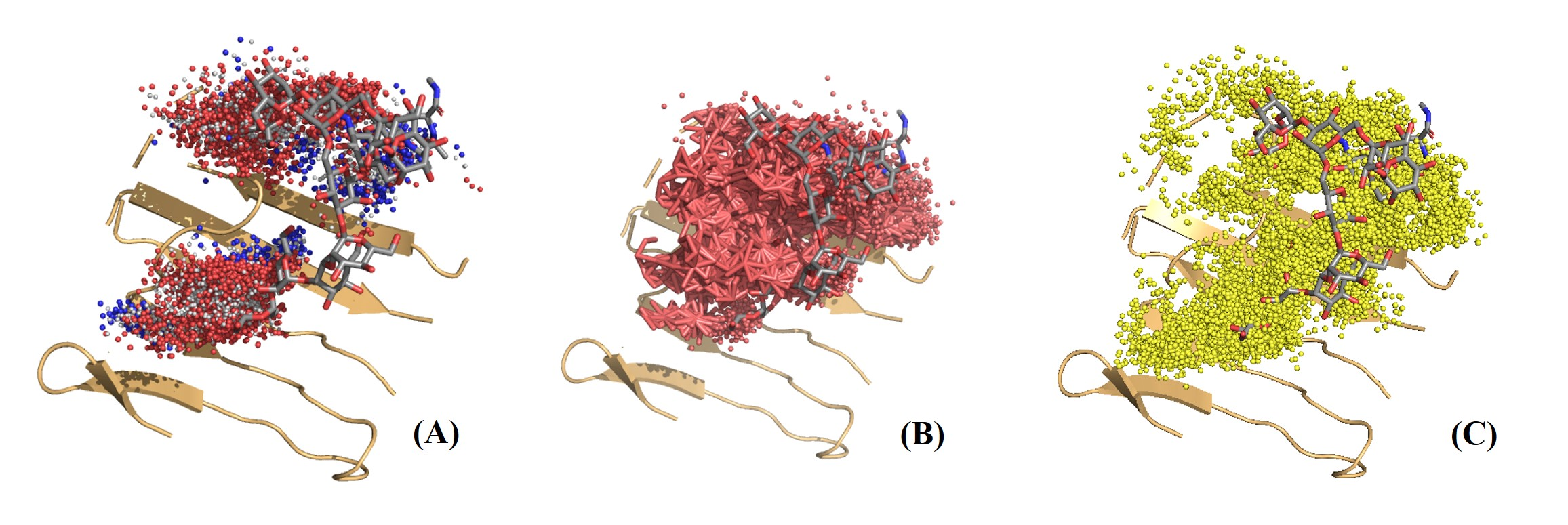

Supplement: S22 Fig — Molecular interaction fields of Calreticulin in Entamoeba histolytica (CRTEh) with monoglucosylated-N-glycan: (A) Hydrogen bond interactions; (B) Hydrophobic interactions; and (C) van der Waal contacts. (TIF) [file pcbi.1010661.s024.tif]

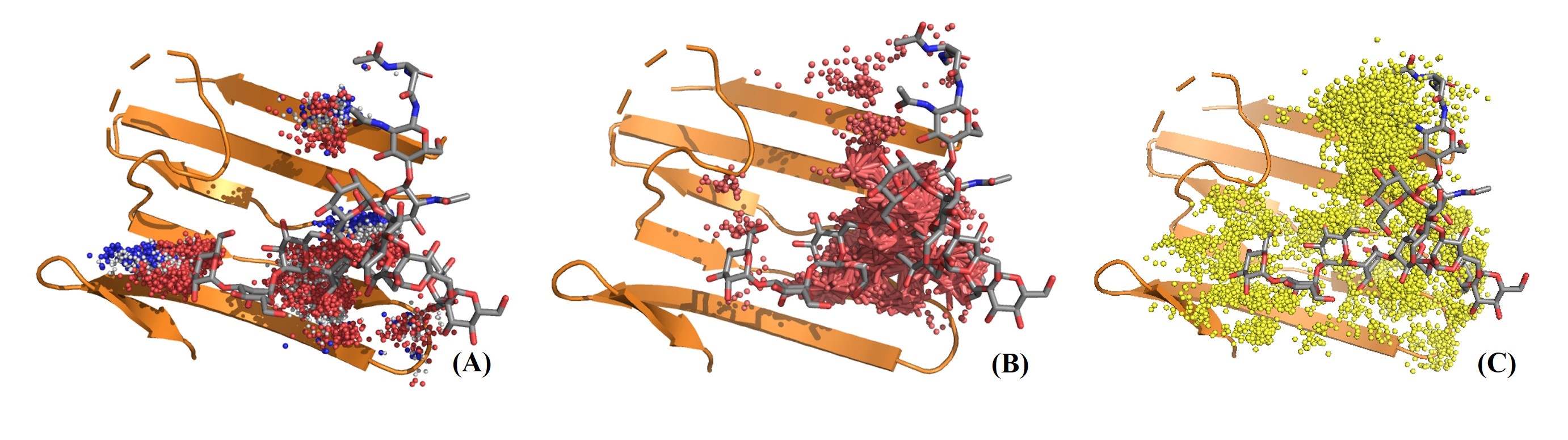

Supplement: S23 Fig — Molecular interaction fields of Calreticulin in Trypanosoma cruzi (CRTTc) with monoglucosylated-N-glycan: (A) Hydrogen bond interactions; (B) Hydrophobic interactions; and (C) van der Waal contacts. (TIF) [file pcbi.1010661.s025.tif]

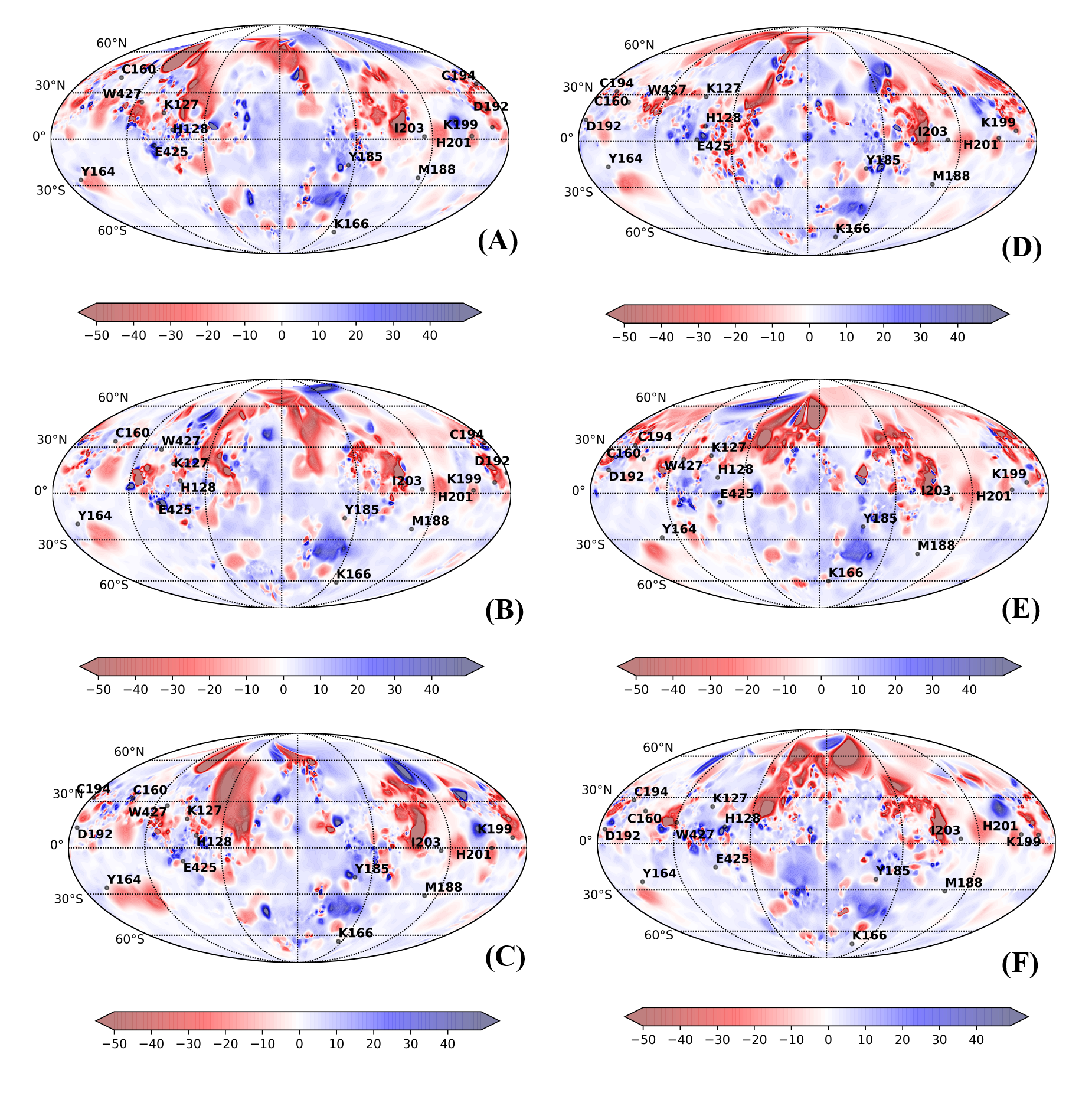

Supplement: S24 Fig — Protein surface topography showing the MEPs mapped for the free and bound conformers of Calnexin in Humans (CNXH): (A) Free-Clus1; (B) Free-Clus2; (C) Free-Clus3; (D) Bound-Clus1; (E) Bound–Clus2; and (C) Bound–Clus3. (TIF) [file pcbi.1010661.s026.tif]

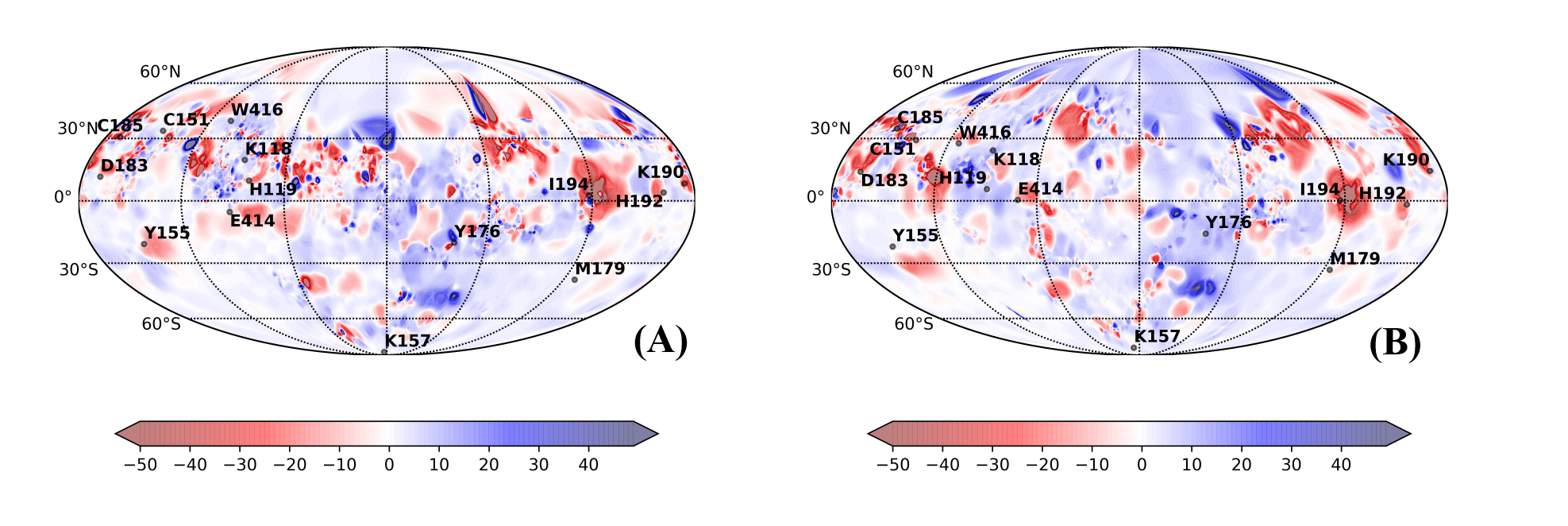

Supplement: S25 Fig — Protein surface topography showing the MEPs mapped for the free and bound conformers of Calmegin in Humans (CLMG): (A) Free-Clus1 and (B) Bound-Clus1. (TIF) [file pcbi.1010661.s027.tif]

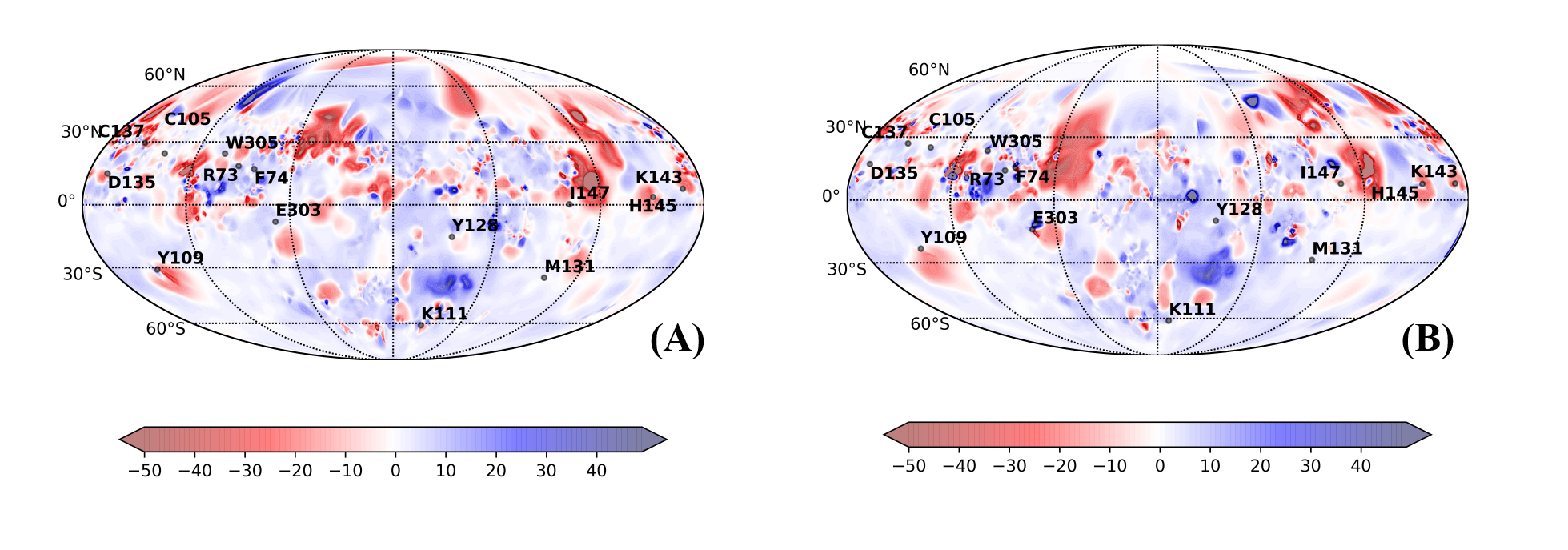

Supplement: S26 Fig — Protein surface topography showing the MEPs mapped for the free and bound conformers of Calsperin in Humans (CALR3): (A) Free-Clus1 and (B) Bound-Clus1. (TIF) [file pcbi.1010661.s028.tif]

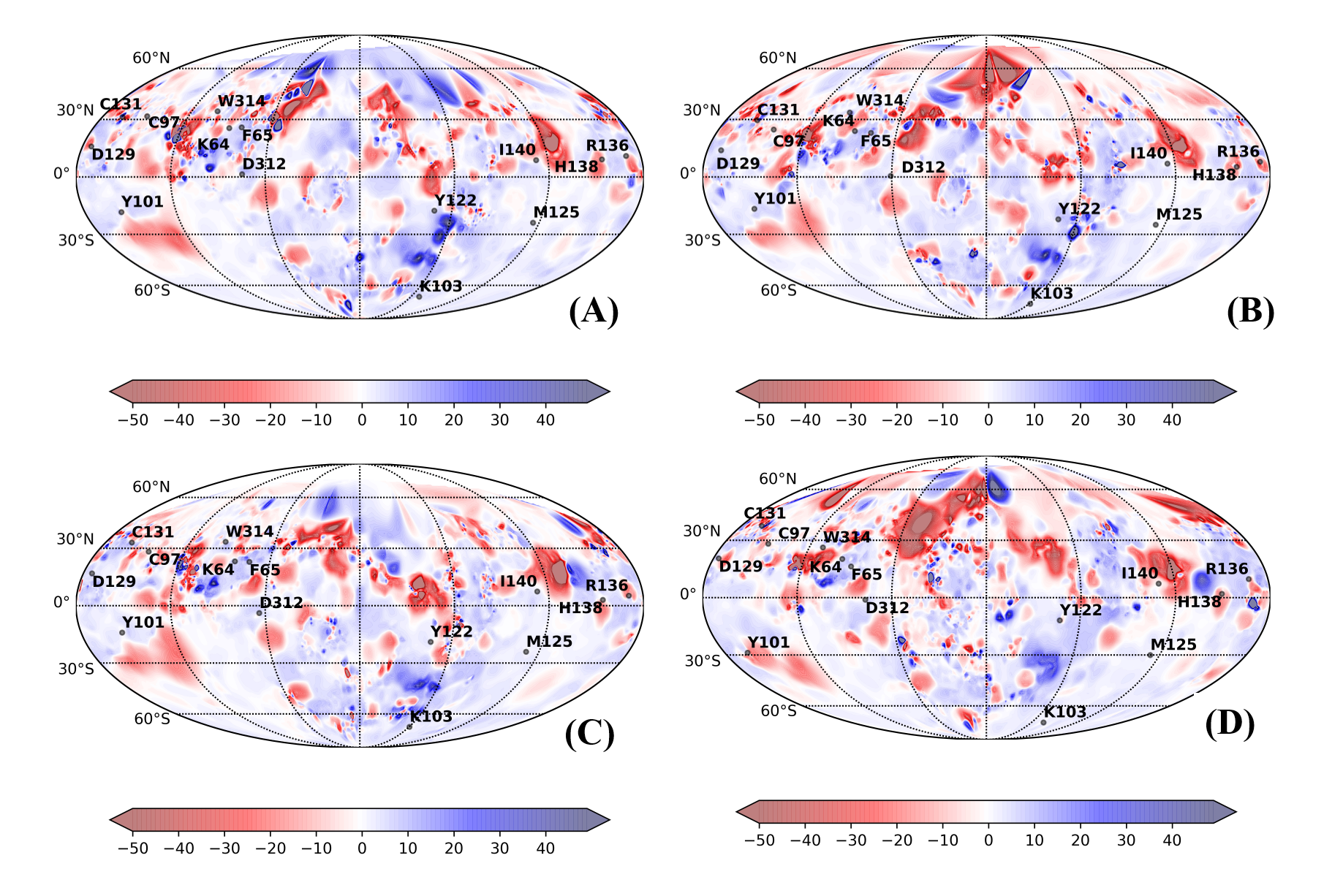

Supplement: S27 Fig — Protein surface topography showing the MEPs mapped for the free and bound conformers of Calreticulin in Entamoeba histolytica (CRTEh) (A) Free-Clus1; (B) Free-Clus2; (C) Free-Clus3; and (D) Bound-Clus1. (TIF) [file pcbi.1010661.s029.tif]

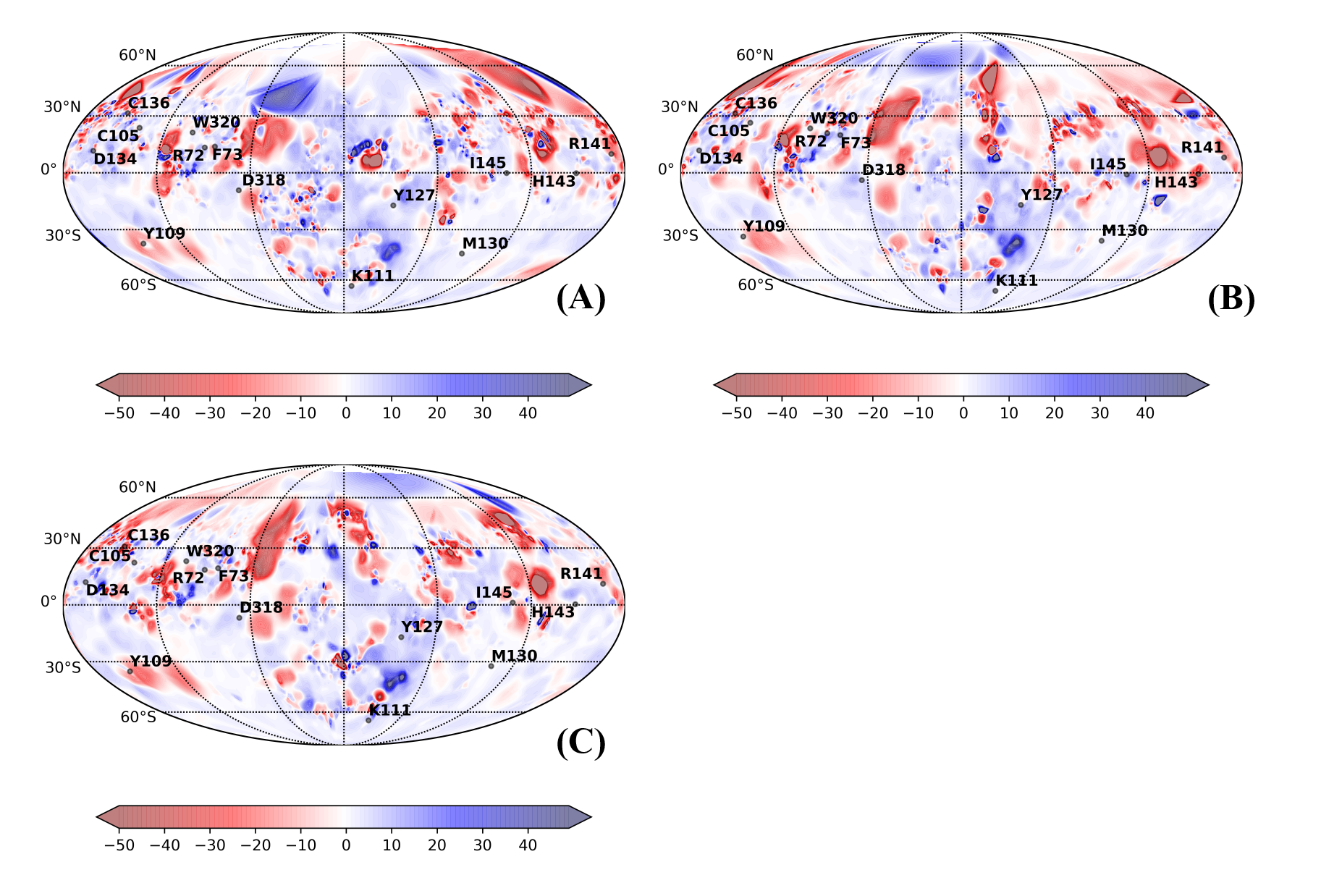

Supplement: S28 Fig — Protein surface topography showing the MEPs mapped for the free and bound conformers of Calreticulin in Trypanosoma cruzi (CRTTc) (A) Free-Clus1; (B) Free-Clus2; and (C) Bound-Clus1. (TIF) [file pcbi.1010661.s030.tif]

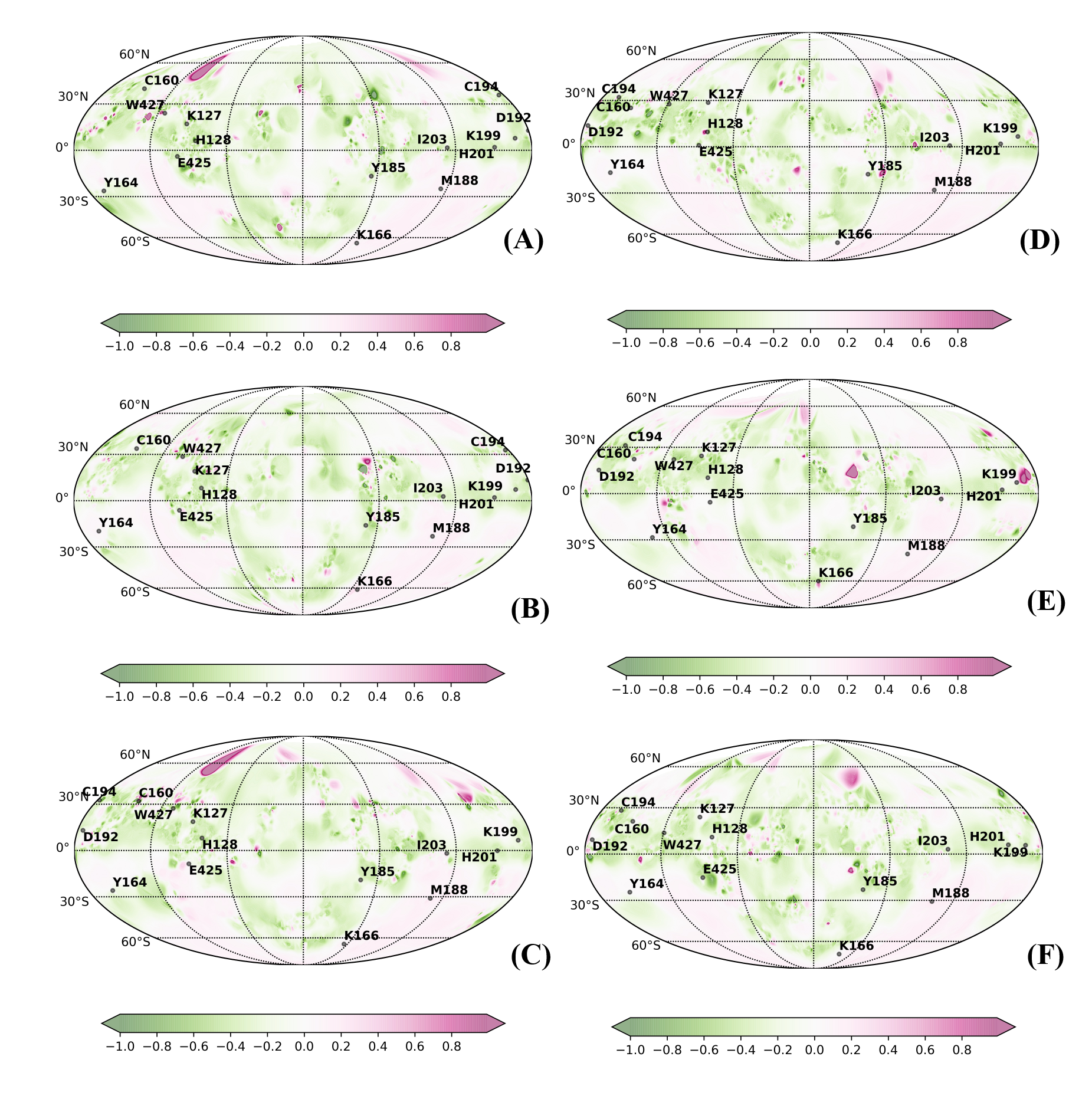

Supplement: S29 Fig — Protein surface topography showing the MHPs mapped for the free and bound conformers of Calnexin in Humans (CNXH): (A) Free-Clus1; (B) Free-Clus2; (C) Free-Clus3; (D) Bound-Clus1; (E) Bound–Clus2; and (C) Bound–Clus3. (TIF) [file pcbi.1010661.s031.tif]

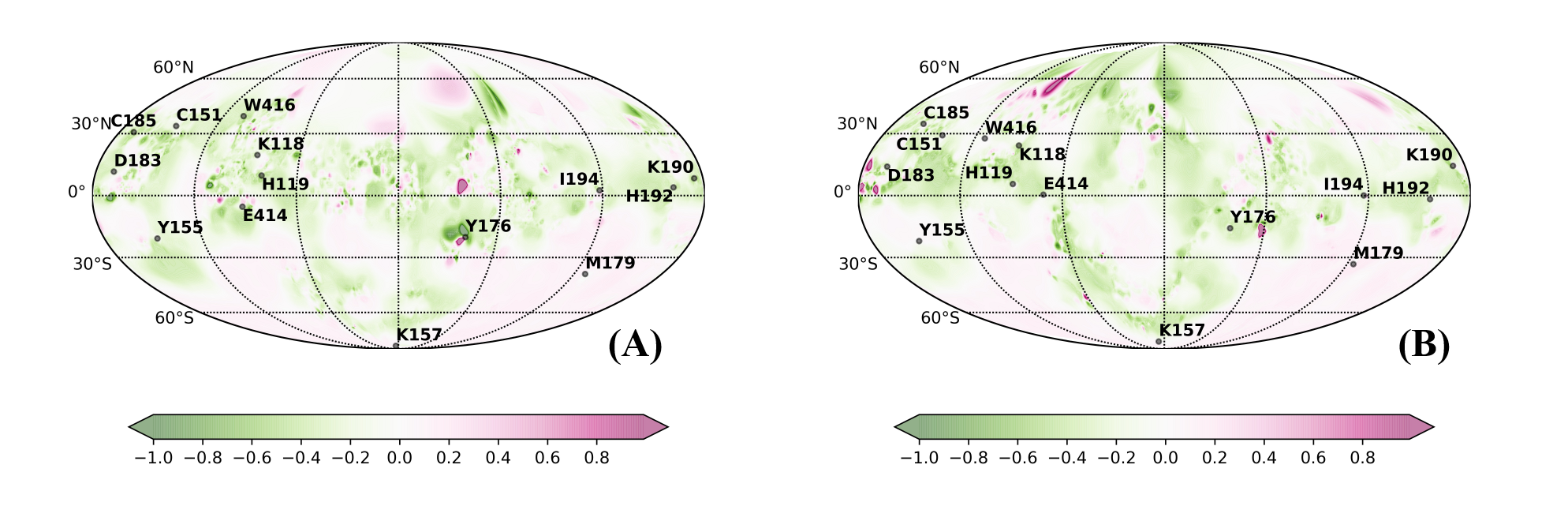

Supplement: S30 Fig — Protein surface topography showing the MHPs mapped for the free and bound conformers of Calmegin in Humans (CLMG): (A) Free-Clus1 and (B) Bound-Clus1. (TIF) [file pcbi.1010661.s032.tif]

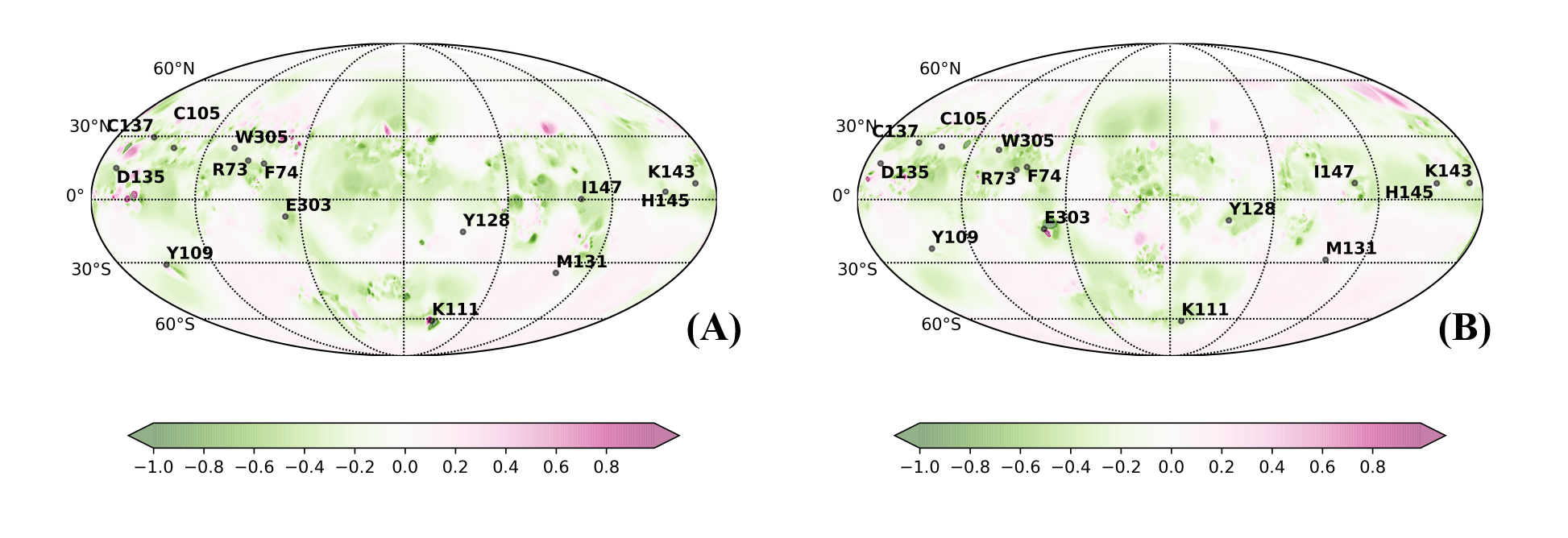

Supplement: S31 Fig — Protein surface topography showing the MHPs mapped for the free and bound conformers of Calsperin in Humans (CALR3): (A) Free-Clus1 and (B) Bound-Clus1. (TIF) [file pcbi.1010661.s033.tif]

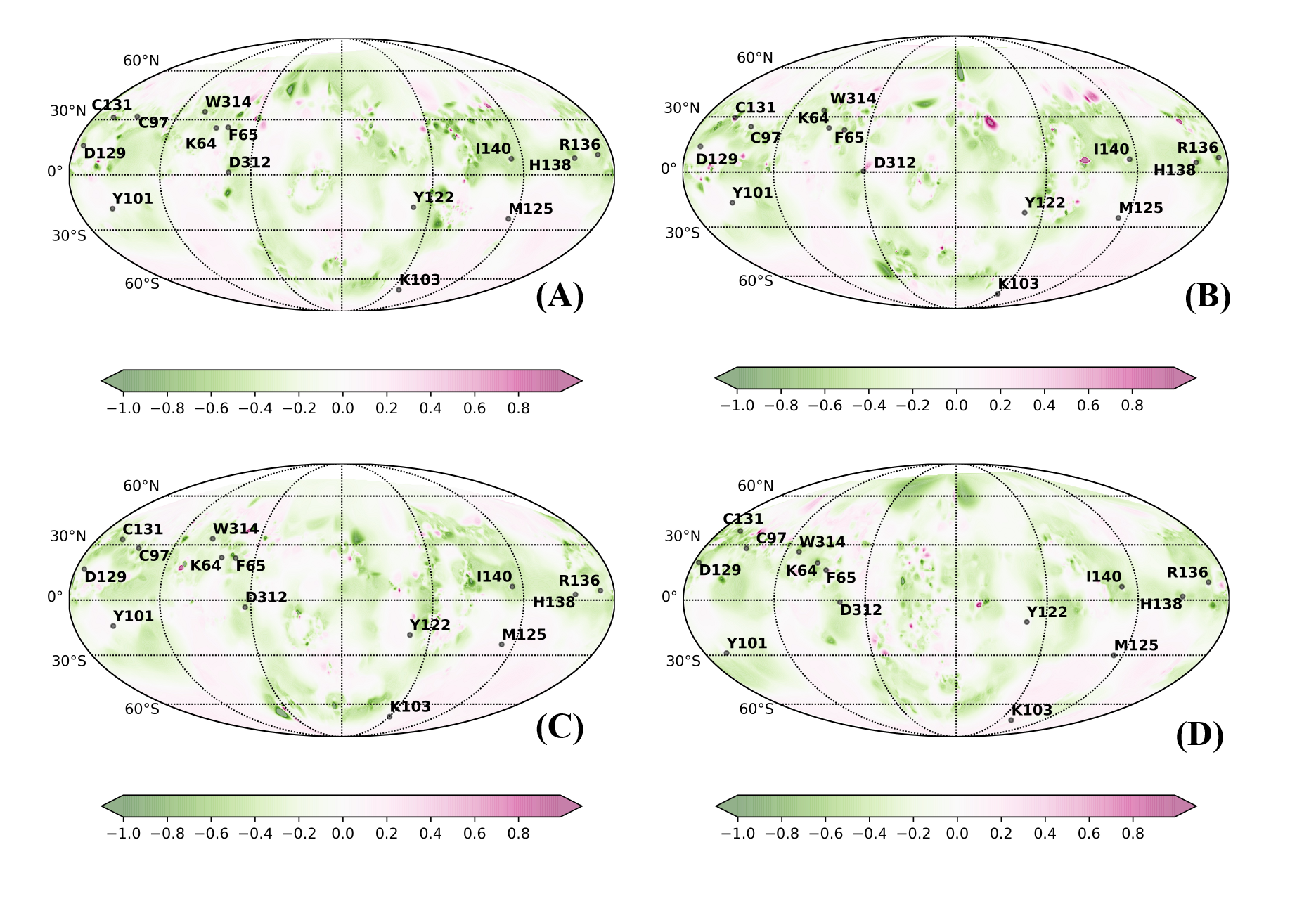

Supplement: S32 Fig — Protein surface topography showing the MHPs mapped for the free and bound conformers of Calreticulin in Entamoeba histolytica (CRTEh) (A) Free-Clus1; (B) Free-Clus2; (C) Free-Clus3; and (D) Bound-Clus1. (TIF) [file pcbi.1010661.s034.tif]

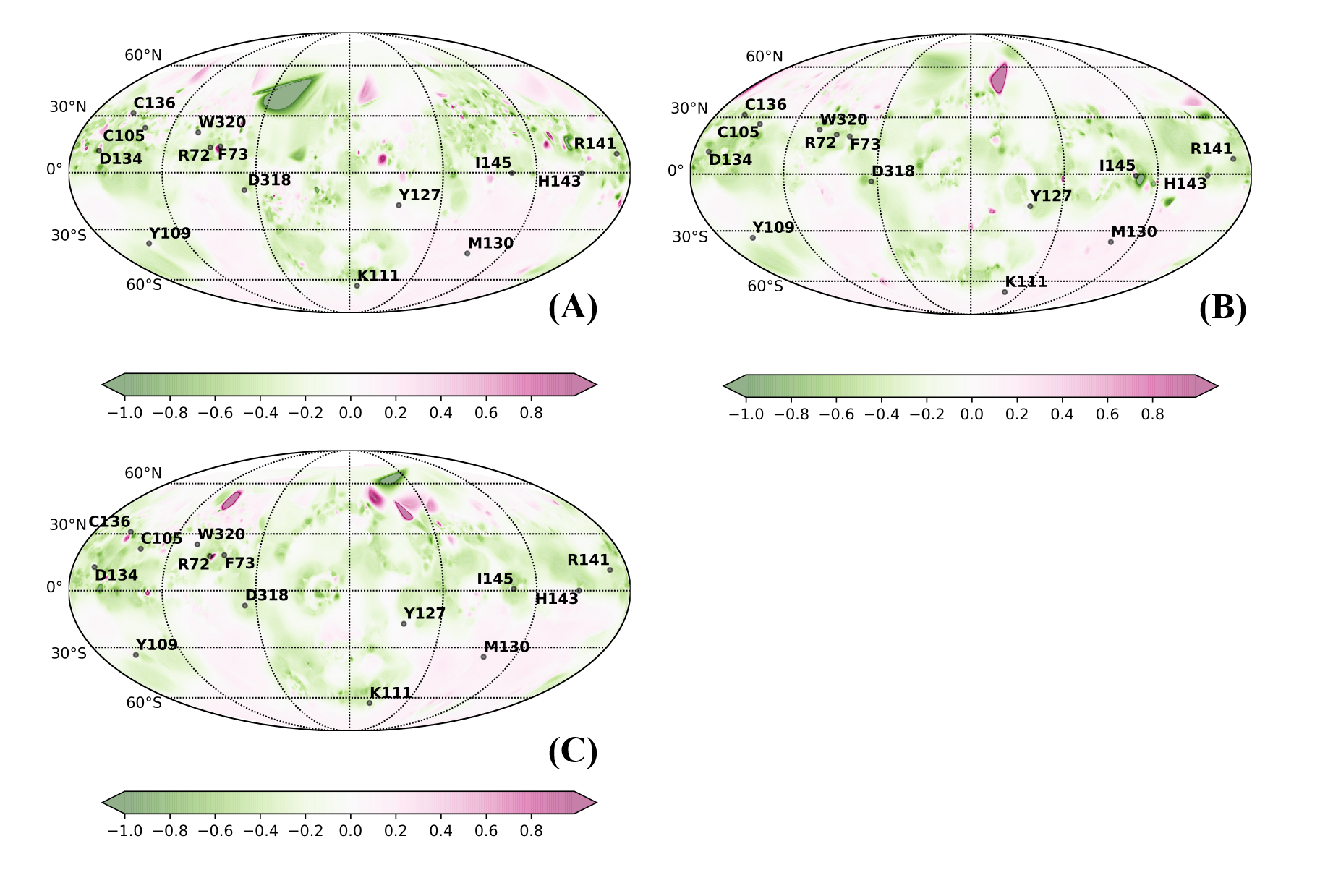

Supplement: S33 Fig — Protein surface topography showing the MHPs mapped for the free and bound conformers of Calreticulin in Trypanosoma cruzi (CRTTc) (A) Free-Clus1; (B) Free-Clus2; and (C) Bound-Clus1. (TIF) [file pcbi.1010661.s035.tif]
